# Structural and mechanistic insights into the cleavage of clustered *O*-glycan patches-containing glycoproteins by mucinases of the human gut

**DOI:** 10.1038/s41467-022-32021-9

**Published:** 2022-07-26

**Authors:** Víctor Taleb, Qinghua Liao, Yoshiki Narimatsu, Ana García-García, Ismael Compañón, Rafael Junqueira Borges, Andrés Manuel González-Ramírez, Francisco Corzana, Henrik Clausen, Carme Rovira, Ramon Hurtado-Guerrero

**Affiliations:** 1 Institute of Biocomputation and Physics of Complex Systems, University of Zaragoza, Mariano Esquillor s/n, Campus Rio Ebro, Edificio I+D, Zaragoza, Spain; 2 Departament de Química Inorgánica i Orgánica (Secció de Química Orgánica) and Institut de Química Teorica i Computacional (IQTCUB), Universitat de Barcelona, 08028 Barcelona, Spain; 3 Copenhagen Center for Glycomics, Department of Cellular and Molecular Medicine, University of Copenhagen, Copenhagen, Denmark; 4 Departamento de Química, Universidad de La Rioja, Centro de Investigación en Síntesis Química, E-26006 Logroño, Spain; 5 Departamento de Biofísica e Farmacologia, Instituto de Biociências, Universidade Estadual Paulista (UNESP), Botucatu, Brazil; 6 Institució Catalana de Recerca i Estudis Avancats (ICREA), 08010 Barcelona, Spain; 7 Fundación ARAID, 50018 Zaragoza, Spain

**Keywords:** X-ray crystallography, Glycoconjugates, Enzyme mechanisms, Glycosylation

## Abstract

Mucinases of human gut bacteria cleave peptide bonds in mucins strictly depending on the presence of neighboring *O*-glycans. The *Akkermansia muciniphila* AM0627 mucinase cleaves specifically in between contiguous (bis) *O*-glycans of defined truncated structures, suggesting that this enzyme may recognize clustered *O*-glycan patches. Here, we report the structure and molecular mechanism of AM0627 in complex with a glycopeptide containing a bis-T (Galβ1-3GalNAcα1-*O*-Ser/Thr) *O*-glycan, revealing that AM0627 recognizes both the sugar moieties and the peptide sequence. AM0627 exhibits preference for bis-T over bis-Tn (GalNAcα1-*O*-Ser/Thr) *O*-glycopeptide substrates, with the first GalNAc residue being essential for cleavage. AM0627 follows a mechanism relying on a nucleophilic water molecule and a catalytic base Glu residue. Structural comparison among mucinases identifies a conserved Tyr engaged in sugar-π interactions in both AM0627 and the *Bacteroides thetaiotaomicron* BT4244 mucinase as responsible for the common activity of these two mucinases with bis-T/Tn substrates. Our work illustrates how mucinases through tremendous flexibility adapt to the diversity in distribution and patterns of *O*-glycans on mucins.

## Abstract (web summary)

AM0627 is a bis-*O*-glycan mucinase that might work in the final steps of mucus degradation, thereby providing a carbon and nitrogen source for *Akkermansia muciniphila*. Here, the authors provide molecular insights into AM0627 function from X-ray crystallography and computer simulations.

## Introduction

**Fig. 1:**
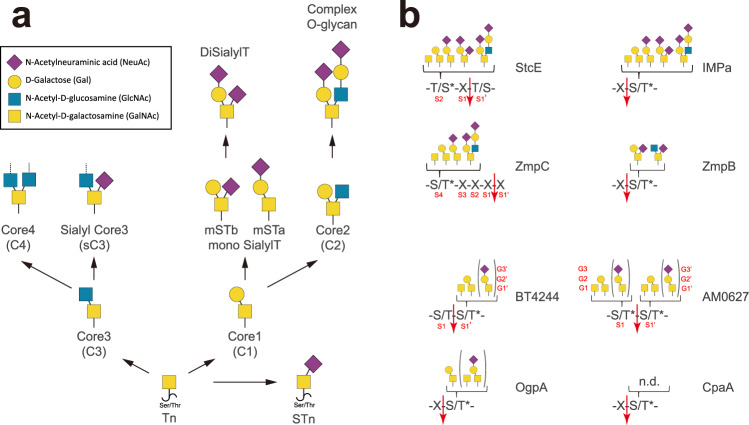
Summary of mucinases/*O*-glycoproteases cleavage motif. **a** The *O*-glycosylation biosynthetic pathway. The GalNAc, Gal, GlcNAc, and Sia are represented as a yellow square, a yellow circle, a blue square, and a magenta diamond, respectively, and according to the symbol nomenclature for glycans (SNFG) described for these monosaccharides^60^. **b** Cleavage motifs for the mucinases and *O*-glycoproteases described in the previous literature. The accepted subsite nomenclature for the amino acids and the sugar moieties of the glycoprotein substrates are indicated for StcE, BT4244, AM0627, and ZmpC as representative examples of these enzymes, and the red arrow indicates the cleaved point. Parentheses  indicate less favored *O*-glycan structures and n.d. indicates that the glycan specificity is not determined.

Mucins are a family of large heavily *O*-glycosylated proteins with tandem repeated sequences (TRs) containing a high proportion of threonines and serine residues serving as *O*-glycan attachment sites^1^. Mucin TRs undergo GalNAc-type *O*-glycosylation (hereafter simply *O*-glycosylation) by a large family of polypeptide GalNAc-transferases^2^ and *O*-glycosylation is one of the most abundant and diverse types of posttranslational modifications (PTMs)^3^. The mucin TR domains, which are densely covered with *O*-glycans^4^, form a rigid bottlebrush-like structure that is largely resistant to general degradation by traditional proteases^1,5^. Mucins line all mucosal surfaces and are the major macromolecules in body fluids serving essential functions in clearance, containment, feeding, orienting, and continuously replenishing our microbiomes and selecting for commensals to repress pathogenic microorganisms^1^. Changes in expression and glycosylation of mucins are associated with human diseases including cancer, and the mucins MUC1, MUC4, and MUC16 serve as circulating biomarkers of different types of cancers^6,7^. Mucins of cancer cells are found with truncated or aberrant *O*-glycans, well-known as specific human tumor-associated carbohydrate antigens (TACAs)^8,9^, while more elaborated or complex *O*-glycans are present in healthy tissues^10^. Among TACAs, the Tn (GalNAcα1-*O*-Ser/Thr), the T (Galβ1-3GalNAcα1-*O*-Ser/Thr), and STn (Neu5Acα2-6GalNAcα1-*O*-Ser/Thr) antigens stand out as the most prevalent and expression of these epitopes are thought to promote tumorigenesis and metastasis^11,12^ (Fig. 1a).

The large gel-forming mucins form oligomeric networks or extended bundles through disulfide bridging in their C/N-termini as part of mucus layers to provide protection for epithelial cells by limiting activation of inflammatory cascades and bacterial contact^13,14^. In the intestine, cross-linked networks of mucin MUC2 form a dense mucin layer that serves as a barrier for microorganisms and a loose mucus layer that entraps and contains the microbiome^1^. In the airways, mucins MUC5B with MUC5AC form long thick bundles that sweep surfaces by cilia movements^15^. Most gut bacteria use dietary fibers and starches as a nutrient source, while a subset of these species can also digest and metabolize host glycans and mucins/glycoproteins^16^. The degradation of mucins is achieved by the combinatorial action of glycosidases^17^, proteases, and an emerging class of so-called mucinases^4^. While the biosynthesis and structures of mucin *O*-glycans are well understood^2,3,18^, the degradation of mucins and in particular their mucin TR domains by bacterial glycoside hydrolases and proteases is still not fully explored^4^. The study of bacterial mucinases capable of cleaving the protein backbone of mucin TR domains densely covered with *O*-glycans is a rapidly evolving field. These mucinases are important for the continuous renewal process and homeostasis of the mucus layers, but they are also used by pathogens to degrade the mucin layers and invade the mucus and reach the underlying epithelium. Understanding the structure and molecular mechanisms of microbial mucinases in the human gut and their substrate specificities is therefore of utmost importance.

Although the definition of mucinases is vague in the literature, they may be considered as a subclass of *O*-glycoproteases that serve a more limited number of substrates such as mucins and mucin domain-containing glycoproteins^4,19^ in comparison to general *O*-glycoproteases, such as *A. muciniphila* OgpA^20^ and *Acinetobacter* CpaA^21^ that target all types of *O*-glycoproteins. Moreover, mucinases may contain separate mucin-binding modules^5,22^, such as the X409 module identified on the *Escherichia coli* O157:H7 StcE mucinase^5^, which is believed to drive StcE mucinase to its mucin substrates. Bacterial *O*-glycoproteases and mucinases mostly cleave glycopeptides N-terminally to contiguous glycosylated Ser or Thr residues (e.g. *B. thetaiotaomicron* BT4244 and *A. muciniphila* AM0908^19^), with exceptions for StcE^19,23^ and *Streptococcus pneumoniae* ZmpC^19,22^. These last two enzymes cleave glycopeptides C-terminally to glycosylated Ser/Thr residues that are near but not contiguous to the cleavage point (Fig. 1b). Most mucinases appear to recognize part of the *O*-glycan(s) adjacent to the *O*-glycopeptide cleavage site^4^. Interestingly the mucinases characterized to date have different tolerance for the structure of the *O*-glycans guiding the peptide bond cleavage, with several mucinases accepting only truncated *O*-glycans such as the simple Tn and T structures without sialic acid capping^4^, while for example StcE functions better with elaborated core 1 and core 2 *O*-glycan structures and blocked by α2–6-linked sialic acid in the core 1 di-sialyl-T (dST) *O*-glycans as well as by core 3 *O*-glycans^5^ (see Fig. 1a, b for the structures of some of these *O*-glycans). These differences in *O*-glycan substrate specificities are likely related to their biological roles. Mucinases from commensals may participate in foraging mucins after their *O*-glycans have been trimmed by exoglycosidases including sialidases to regulate normal mucus homeostasis. However, mucinases like StcE from invading pathogens may instead participate in the pathogenesis by destroying the most nascent mucins with intact *O*-glycans and penetrate the dense mucus layer that protects the microbiome from reaching the epithelium. Mucinases are expressed by commensal bacteria such as *B. thetaiotaomicron* (BT4244), *A. muciniphila* (AM0908, AM1514 and AM0627), and pathogenic bacteria such as *Clostridium perfringens* (ZmpB), *Pseudomonas aeruginosa* (IMPa), *S. pneumoniae* (ZmpC), *E. coli* O157:H7 (StcE), and enteroaggregative *E. coli* (Pic)^4^. So far only StcE and ZmpC have been shown to cleave glycopeptides containing complex *O*-glycans^19,22,23^ (Fig. 1a, b). Mucinases and *O*-glycoproteases appear to be promiscuous in terms of substrate peptide sequence specificity^4^, although refinement of substrate peptide sequences is still in progress^24^.

While most of the enzymes listed above depend on a single *O*-glycan for cleavage of *O*-glycopeptides, *A. muciniphila* AM0627 was recently described to require two adjacent truncated *O*-glycans and cleave in between the two *O*-glycosites^19^ (Fig. 1b; see also the accepted subsite nomenclature for the amino acids and the sugar moieties of the glycoprotein substrates). Very recently, a crystal structure of the Zn^2+^-bound AM0627 (residues 21–506; PDB entry 7SCI) was reported^25^, but the molecular/structural basis for the bis-*O*-glycan substrate requirement was not elucidated.

Here, we applied a multidisciplinary approach encompassing biophysical and computational techniques to reveal the molecular basis for catalysis and *O*-glycopeptide selectivity by AM0627. We report the structure of AM0627 in complex with a bis-T glycopeptide and demonstrate that the enzyme interacts with both glycans in the bis-*O*-glycopeptide, preferentially recognizing the Galβ1-3GalNAc disaccharide (T *O*-glycan) at the G1 and G2 subunits and the GalNAc sugar (Tn *O*-glycan) at the G1’ subunit (Fig. 1b; see also the accepted subsite nomenclature for the sugar moieties). Moreover, we uncover the enzyme catalytic mechanism by QM/MM simulations, showing that a nucleophilic water and a catalytic base Glu residue are required for efficient catalysis. We demonstrate, using well-defined mucin reporter substrates, that AM0627 prefers T over Tn *O*-glycan substrates and is essentially inactive with sialylated complex *O*-glycosylated substrates. Finally, we identify a Tyr residue in AM0627 that is conserved in BT4244, which participates in a key interaction with the T *O*-glycan at S1 and provides a basis for the bis-*O*-glycan substrate specificity of AM0627 and likely also BT4244 mucinases.

## Results

### Architecture of the AM0627–bis–T glycopeptide–Zn^2+^ complex

**Fig. 2:**
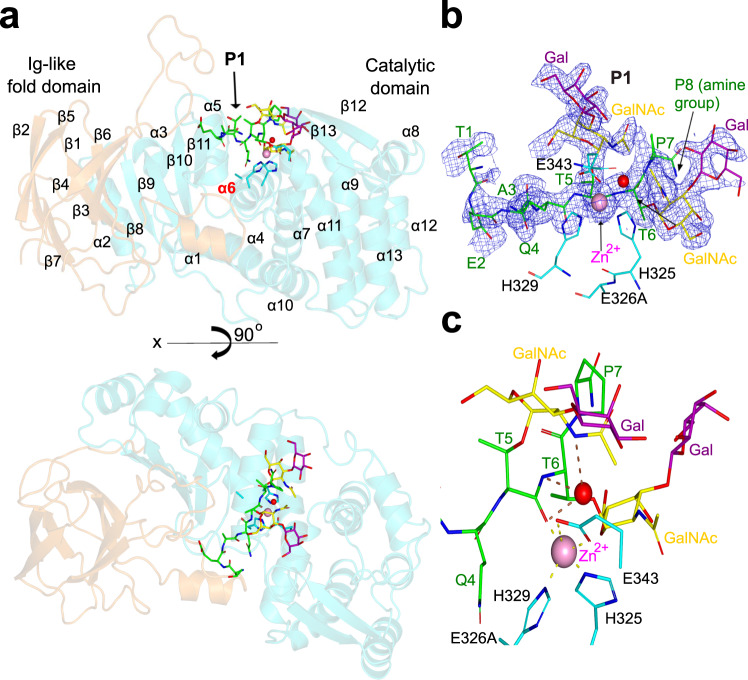
Crystal structure of the AM0627^E326A^–P1–Zn^2+^ complex. **a** Ribbon structure of AM0627^E326A^ complexed to **P1** and Zn^2+^ (upper panel). In the lower panel, the monomeric form is displayed in a different view. The IgG fold and the catalytic domains are colored in orange and cyan, respectively. The secondary structures are only indicated in the upper panel and α6 is highlighted in red. The **P1** amino acids are depicted with green carbon atoms whereas the GalNAc and Gal are shown as yellow and magenta carbon atoms, respectively. While the GalNAc moieties are shown in yellow according to the SNFG^60^, the Gal moieties are shown in magenta for illustration purposes. The Zn^2+^ and a water molecule are shown as a pink and red sphere, respectively. **b** Close-up view of the active site showing the bound Zn^2+^, a water molecule, and **P1**. His325, Ala326, His329, and Glu343 are shown as cyan sticks. Electron density maps are Fo–Fc (blue) contoured at 2.2*σ* for **P1** and 2Fo-Fc (blue) contoured at 1*σ* for Zn^2+^ and a water molecule. Only the residues of **P1** with well-defined density (Ala3 to Thr6) and partial density are displayed (Thr1, Glu2, Pro7, and the amine group of Pro8). **c** Close-up view of the active site showing the pentagonal geometry formed by the Zn^2+^ and the coordinating residues (see dotted yellow lines for the interactions between the metal and the amino acids). The hydrogen bonds between the water molecule and **P1** are indicated as dotted brown lines.

To gain insights into the structure of AM0627 and its recognition and cleavage of bis-*O*-glycans-containing glycopeptides, we pursued the determination of its structure by X-ray crystallography. Initially, we chemoenzymatically synthetized a P-selectin glycoprotein ligand 1 (PSGL-1)-like bis-T glycopeptide (hereafter **P1** and defined by the sequence TEAQT**T**PPPA in which ** denotes a Galβ1-3GalNAc disaccharide) based on AM0627 substrate cleavage consensus motif determined by a previous mass spectrometry study^19^. We designed a first construct that did not contain the predicted signal sequence and mutated the putative catalytic Glu326 to Ala in order to express an inactive form of AM0627 in *E. coli*, as reported before^19^ (residues A21-E506; see Supplementary Fig. 1a and see the “Methods” section). Despite crystals appeared, these diffracted poorly and prompted us to design a shorter construct of the E326A-inactive mutant that started in Pro71 and finished in Glu506 (hereafter AM0627^E326A^). We predicted that the first 50 residues (Ala21–Lys70) were comprised of several α-helices and loops that were well separated from the catalytic domain, which was confirmed by the Zn^2+^-bound AM0627 crystal structure (residues 21–506; PDB entry 7SCI) reported^25^. The truncated AM0627^E326A^ (P71-E506) enzyme construct produced better diffracting orthorhombic crystals (space group P2_1_2_1_2_1_) in the presence of **P1** and ZnCl_2._ We, therefore, tested if the truncated AM0627 (AM0627^P71-E506^) was active and comparable in activity to the wild-type (wt) AM0627^A21-E506^ enzyme using an artificial Tn bis-*O*-glycan reporter based on our previously reported mucin reporter design^5^. This reporter contains multiple 12-mer repeats with bis-*O*-glycosites (AEAAA**TT**PAPAK)_*n*=18_, and the Tn *O*-glycoform of this reporter was produced homogeneously in HEK293 cells with KO of *C1GALT1*. The wt AM0627^A21-E506^ and the AM0627^P71-E506^ exhibited similar efficient cleavage of this *O*-glycan substrate when decorated by Tn *O*-glycans (Supplementary Fig. 1b), confirming that the truncated AM0627^P71-E506^ represented a fully active enzyme suitable for structural studies. The crystal structure of AM0627^E326A^ was solved at 1.9 Å using zinc single-wavelength anomalous dispersion (Zn-SAD; see the “Methods” section). The model obtained from SHELXE^26^ allowed us to solve the structure of the AM0627^E326A^–**P1**–Zn^2+^ complex at a higher resolution (1.5 Å) by molecular replacement using PHASER^27^ (see Supplementary Table 1 and see the “Methods” section). Although the asymmetric unit (AU) of P2_1_2_1_2_1_ crystals contained two molecules of AM0627^E326A^ that partially contacted each other, gel filtration chromatography (Supplementary Fig. 2) showed that AM0627^E326A^ was monomeric, which was further confirmed by the PISA server^28^. The AM0627^E326A^ crystal structure revealed two distinct domains formed by an Ig-like fold domain and the M60-like catalytic domain (Fig. 2a). Ig-like fold domains were also found in previous mucinases crystal structures such as the ones reported for BT4244, ZmpB and IMPa^29^. With respect to the M60-like catalytic domain, BT4244 and ZmpB structures share a similar M60-like catalytic domain^29^ which, according to MEROPS database^30^, is also present in AM0908^4^. The root-mean-square deviation (RMSD) between both molecules belonging to chains A and B in the AU is 0.44 Å on 437 equivalent Cα atoms. Hereafter we will discuss only molecule B because it contains a better-defined density for **P1** and particularly for the sugar moieties (Fig. 2b). In addition, the AM0627 mucinase contained the HEXXH motif^4^, formed by His325, Glu326 (in the AM0627^E326A^ structure, Glu326 was mutated to Ala326) and His329 (Fig. 2b) and located in a conserved alpha helix (α6) (Fig. 2a). The equivalent alpha helix in OgpA and other mucinases was recently shown to be variable in length and suggested to underlie the difference in substrate specificity between these enzymes^20^. An additional conserved Glu residue (Glu343 in AM0627) together with the two His residues of the HEXXH motif and the **P1** Thr5 carbonyl group coordinate the zinc ion to form a pentagonal geometry (Fig. 2c), a feature that fits with AM0627 belonging to the gluzincin-like family of zinc metallopeptidases^4^. The zinc ion in the Zn^2+^-bound AM0627 crystal structure was only coordinated in a trigonal geometry by His325, His329, and Glu343^25^. In addition, a water molecule did not coordinate the zinc ion and therefore did not replace the role of the **P1** Thr5 carbonyl group in coordinating the metal. However interestingly, in our structure, we observed the presence of a potential catalytic water molecule, further discussed below, which was visualized in the active site establishing hydrogen bonds with the Thr5 carbonyl group, the acetamide NH group of the GalNAc located at G1, and the NH group of Thr6 (Fig. 1b for the subsite nomenclature of the sugar moieties and Fig. 2b, c).

### AM0627 recognizes bis-T *O*-glycans within a specific peptide sequence

**Fig. 3:**
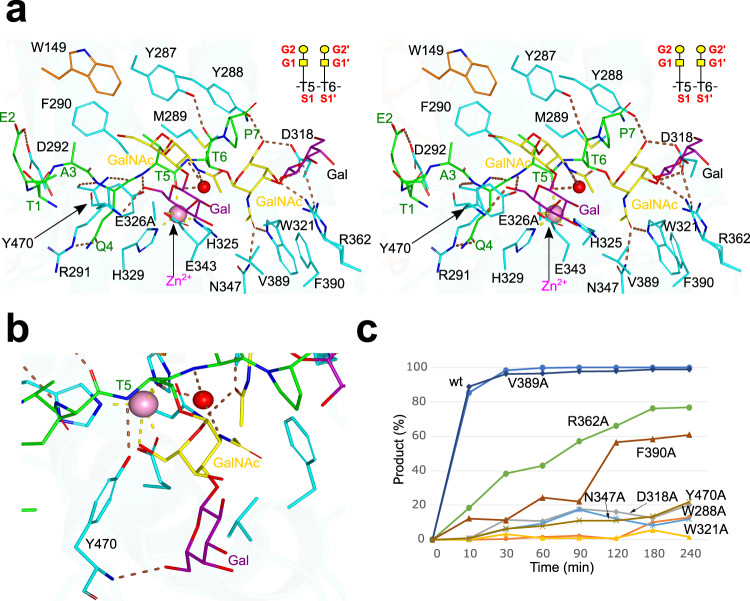
Structural features of the active site. **a** Stereo view of the active site for the AM0627^E326A^–**P1**–Zn^2+^ complex. The residues forming the active site are depicted as cyan (catalytic domain) and orange (IgG-fold domain) carbon atoms. The rest of the colors for the metal, the water molecule, and the dotted lines for the hydrogen bond interactions are the same as described in Fig. 2 . The GalNAc moieties are displayed in yellow according to the SNFG^60^, while the Gal moieties are displayed in magenta for illustration purposes. The inset shows a scheme of the subsite nomenclature for the amino acids and the sugar moieties of **P1**. Note that the sugar moieties in the inset are displayed in yellow and with different symbols according to the SNFG. **b** Alternative close-up view of the active site to highlight the interactions between Tyr470 and the GalNAc and Gal moieties located at G1 and G2, respectively. **c** Time course of a cleavage assay using the 0.4 μM of wt AM0627^A21-E506^ and the different mutants with **P1** (57 μM) incubated at 37 °C. The cleavage was monitored by detecting the peak of substrate and product by MALDI spectra and the % of the remaining substrate and product formation was estimated. The raw spectra of MALDI-TOF analysis are given in Supplementary Fig. 3. All experiments were done in duplicate (*n* = 2 independent experiments). Source data are provided as a Source Data file. Note that each time point represents the average of two independent determinations.

The AM0627-active site is formed by the zinc-binding site, discussed above, and the glycopeptide binding site (Fig. 3a). At the level of the peptide sequence of **P1**, the main enzyme–peptide interactions are as follows. The Glu2 side chain makes a hydrogen bond with the Asp292 side chain, the Ala3 methyl group establishes CH–π interactions with the side chains of Trp149/Phe290, and Gln4 forms hydrogen bonds with Arg291 (both side chain and backbone). Regarding the two glycosylated Thr residues (Thr5 and Thr6 in Fig. 3a), the Thr5 backbone forms a hydrogen bond with the Tyr470 side chain, the Thr5 methyl group forms a CH–π interaction with the Tyr287 side chain and the Thr6 backbone makes a hydrogen bond with the Tyr287 side chain (Fig. 3a).

Additional enzyme–substrate interactions involve the two contiguous T *O*-glycans (bis-T) of **P1**. Both the GalNAc and Gal located at G1 and G2 (see inset in Fig. 3a for the accepted subsite nomenclature for the sugar moieties), respectively, establish CH–π interactions with Tyr470. The OH6 group of the Gal at G2 also makes a hydrogen bond with the Tyr470 backbone (Fig. 3b). Interestingly, the GalNAc located at G1’ is the sugar establishing the highest number of interactions with AM0627. The acetamide carbonyl and methyl groups form hydrogen bonds with the side chains of Trp321/Asn347, as well as CH–π interaction with Phe390, respectively, and the GalNAc OH3 and OH6 groups make hydrogen bonds with Asp318/Arg362 and Tyr288/Asp318 side chains, respectively. Finally, the Gal at the G2’ subunit is mostly solvent exposed and poorly recognized, forming only one hydrogen bond between its endocyclic oxygen and Arg362 side chain (Fig. 3a). These interactions show that both the peptide sequence and most of the sugar units of **P1** are well recognized, indicating that AM0627 likely has clear preferences for specific amino acids and the sugar moieties of glycopeptides. In addition, these results suggest that, although mucinases recognize a large variety of peptide sequences within the *O*-glycoproteome, they may also show distinct specificities for the amino acids nearby to the *O*-glycans.

Our analysis of the glycopeptide interaction disagrees somewhat with the recent docking experiments performed with the Zn^+2^-bound AM0627 crystal structure^25^. In this study, the T *O*-glycan at S1’ occupied a similar position to that found for the corresponding glycan of our **P1**. However, the sugar moieties of the T *O*-glycan at S1 were located nearby to Trp149 and Phe290^25^, a completely different environment of the enzyme compared to the one inferred from our crystal structure in which the **P1** T *O*-glycan at S1 interacts with Tyr470 (Fig. 3b). Nevertheless, the previous study, which includes activity analysis of mutant enzymes with Trp149, Tyr287 and Phe290 to Ala residues substitutions with different glycoprotein substrates, confirmed the importance of these residues in peptide recognition^25^, supporting our crystal structure of the AM0627^E326A^–**P1**–Zn^2+^ complex (Fig. 3a). In addition, these mutants were less active than the wt enzyme and also showed different activity profiles towards glycoprotein substrates, validating their role in recognition of the peptide sequences^25^. The previous results did not explain the activity of AM0627 as a bis-*O*-glycan mucinase, but reinforced our conclusion on the role of Trp149, Tyr287, and Phe290 in peptide recognition.

**Fig. 4:**
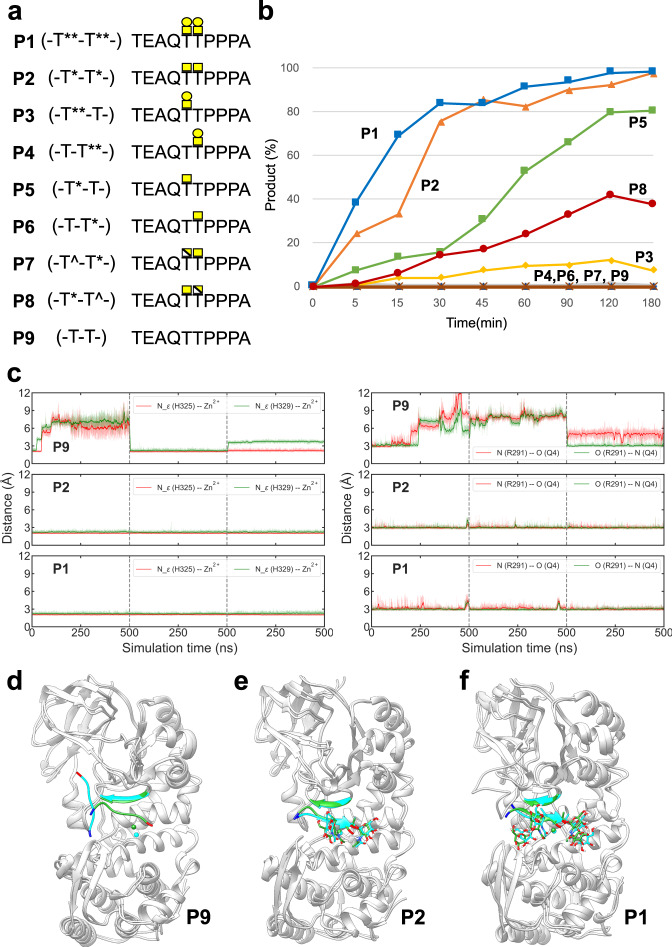
Selectivity of wt AM0627^A21-E506^ for  peptides containing different *O*-glycan forms. **a** Scheme of the different (glyco)peptides used in this study. Note that both the GalNAc and Gal moieties are indicated as a yellow square and circle, respectively, whereas galactosamine is indicated as a white/yellow square. The sugar moieties are displayed in different colored symbols according to the SNFG^60^. **b** Time course of the cleavage assay using the 0.4 μM of wt AM0627^A21-E506^ with the different (glyco)peptides (500 μM) incubated at 37 °C. See the raw spectra of MALDI-TOF analysis in Supplementary Fig. 4. The specific activity of AM0627 was calculated using the **P1** glycopeptide at a time point 5 min where 38% **P1** cleavage took place (rate of conversion 5.7 nmol substrate/360 nmol enzyme/5 min). Note that substrate inhibition might be present for the wt AM0627^A21-E506^ at higher concentrations of **P1**, explaining that the slope is less-steeper for **P1** in Fig. 4 than the slope for **P1** in Fig. 3 (**P1** is at 57 μM in Fig. 3). The concentrations of the (glyco)peptides were higher than that of **P1** in Fig. 3 to figure out whether other (glyco)peptides might be substrates for the wt AM0627^A21-E506^. All experiments were done in duplicate (*n* = 2 independent experiments). Source data are provided as a Source Data file. Note that each time point represents the average of two independent determinations. **c** Evolution of several distances (His324-N_ε_···Zn^2+^, His329-N_ε_···Zn^2+^ and N_R291_···O_Q4_ and N_R291_···N_Q4_) during the MD simulation of AM0627 in complex with **P1**, **P2**, and **P9**. The simulations were performed in triplicate (3 independent experiments). **d**–**f** Overall structure of AM0627 complexed to **P9**, **P2**, and **P1** showing the dynamics of these (glyco)peptides during the MD simulations. The initial positions of the glycopeptides, the β-sheet formed by Met289-Asp292 and Zn^2+^ are shown in green, while the final positions (after one of the 500 ns replicas) are shown in cyan. The N- and C-termini of the glycopeptides are colored in blue and red, respectively. A closer view of **e** and **f** is shown in Supplementary Fig. 5, and more detailed interactions between AM0627 and these (glyco)peptides are shown in Supplementary Figs. 7 and 8.

To get insights into the role of residues of AM0627 engaged in interactions with the sugar units of the **P1** bis-T *O*-glycans, we tested Ala mutations of Tyr288, Asp318, Trp321, Asn347, Arg362, Phe390, and Tyr470, and the resulting mutants were characterized in vitro (see the “Methods” section). As a positive control, we tested the V389A mutant, since Val389 does not interact with **P1**. To generate these mutants, we used the plasmid pMALC2x-12Hist-TEV-*AM0627*^*21–506*^ that encodes for the wt AM0627^A21-E506^, and a time-course assay of digestion of the **P1** glycopeptide was monitored by MALDI-TOF (see the “Methods” section) (Fig. 3c). This assay revealed that the wt enzyme and the V389A mutant cleaved almost 90% of the P1 substrate at the first time-point of 10 min, while most of the deleterious mutants showed <20% cleavage at 10 min. However, after 240 min, only R362A and F309A showed a significant time-dependent increase in cleavage reaching 80% and 60% cleavage, respectively (Fig. 3c and Supplementary Fig. 3). Overall, most of the mutants either displayed poor cleavage or were completely inactive. The time-course MALDI-TOF analysis of the cleavage reaction used in this work does not allow for a more detailed analysis of the kinetic properties as MALDI-TOF is only semi-quantitative and it is challenging to quantify cleavage at low substrate concentrations. Instead, we attempted to assess the highest specific activity obtained with AM0627 using the **P1** glycopeptide and estimated this to be at least 1.9 U/mg (where U is enzyme units and is defined as μmol/min; Fig. 4b). Interestingly, the R362A and F390A mutants showed significant activities, albeit at more than ~30× fold lower cleavage that the wt and V389A enzymes (Fig. 3c), suggesting that Arg362 and Phe390 are not essential for activity. Interestingly, Tyr470, the only residue engaged in interactions with GalNAc and Gal at the G1 and G2 subunits, as well as Tyr288, Asp318, Trp321, and Asn347 (the residues interacting with the GalNAc at the G1’ subunit), were found to be critical for AM0627 activity, suggesting that these residues are the main players in driving recognition towards the sugar units of the bis-T *O*-glycans. These results, as suggested by the structural analysis, are consistent with the poor recognition of AM0627 towards Gal at the G2’ subunit. Overall, the mutagenesis analysis shows that the driving force for AM0627 binding to the bis-T substrate is the first Galβ1-3GalNAc disaccharide at S1 and the GalNAc residue at the G1’ subunit of S1'.

### AM0627 preferably cleaves bis-T over bis-Tn substrates and depends on the GalNAc at G1 as the minimal *O*-glycan structure for activity

AM0627 was recently reported to cleave bis-*O*-glycopeptides containing T *O*-glycans as well as Tn and sialylated core 1 (mSTa) *O*-glycans (Fig. 1b)^19^, and further evidence for this was presented in the recent report^25^. To get further insights into the type of *O*-glycans being recognized and the minimal *O*-glycan structure required for cleavage, we synthetized a battery of glycopeptides containing GalNAc, galactosamine (GalN), and Gal-GalNAc (see Fig. 4a). The synthesis of the glycopeptides with different *O*-glycan positions and structures (**P2–P9**) were based on the **P1** peptide sequence (TEAQTTPPPA). The **P2** glycopeptide contained a bis-Tn while **P5** and **P6** glycopeptides had one single GalNAc moiety at G1 and G1’, respectively. In addition, we synthetized one diglycopeptide containing a GalNAc moiety at G1 and a GalN at G1’ (**P8**), and another one with the inverse order of sugar units (**P7**). The two mono-T glycopeptides **P3** and **P4**, which contain the mono-T *O*-glycan in the S1 and S1’ positions, respectively, were chemoenzymatically synthetized using **P5** and **P6** as templates. A naked peptide, **P9**, was also made to confirm whether cleavage might take place in the absence of the *O*-glycans. The use of GalN in place of GalNAc was considered in order to explore the role of the acetyl group in substrate recognition (Fig. 4a).

The MALDI-TOF time-course assays with the wt AM0627 enzyme and different glycopeptide substrates showed that the **P1** and **P2** glycopeptides with bis-T and bis-Tn *O*-glycans, respectively, served as the best substrates with cleavage of **P1** being slightly faster than that of **P2** at the early time points expected with maximum velocities of reactions (e.g. at 5 min, ~40% **P1** and ~25% **P2** were cleaved while at 15 min, ~70% **P1** and ~35% **P2** were cleaved). Note that the highest specific activity for AM0627 using the **P1** was estimated because under a higher concentration of substrate (500 μM) and short time points (5–30 min), the reaction was almost linear, reaching ~50% cleavage of **P1** at 10 min and 100% cleavage at 120 min. This was likely due to substrate inhibition taking place at a high concentration for **P1** (compare Figs. 3c and 4b). Based on the same rationale described above, the specific activity for the second-best substrate, **P2**, was 1.6-fold lower than that of **P1** (1.2 U/mg for **P2** versus 1.9 U/mg for **P1**). The **P5** mono-Tn (-T*-T-) glycopeptide was a considerably poorer substrate (<10% cleavage at 5 min and <15% at 15 min), followed by **P8** (-T*-T^-) with a modified bis-Tn (<5% cleavage at 5 min and <10% at 15 min) and the **P3** mono-T (-T**-T-) with barely detectable cleavage (<5% at 15 min). The **P4** mono-T (-T-T**-), the **P6** mono-Tn (-T-T*-), and the **P7** glycopeptide (-T^-T*-) were completely resistant to cleavage. Finally, the naked **P9** peptide without *O*-glycans served as the non-substrate control demonstrating that the presence of *O*-glycans was absolutely required for catalysis (Fig. 4b and Supplementary Fig. 4). Comparison of the enzyme activity with **P7** (-T^-T*-) versus **P8** (-T*-T^-) indicated that the acetyl group of the GalNAc at the G1 subunit is indispensable for activity. These results indicate that AM0627 has a preference for glycopeptide substrates with bis-T *O*-glycans compared to bis-Tn *O*-glycans, and only cleaved glycopeptides with one *O*-glycan when the *O*-glycan was positioned in the G1 subunit and not G1’. Moreover, the GalNAc residue at the G1 subunit is essential for cleavage since substituting the GalNAc at G1 with GalN abrogated cleavage (**P7**), while substitution at G1’ only reduced cleavage (**P8**).

To rationalize the above findings at the molecular level, we performed molecular dynamics simulations of enzyme complexes with **P1**, **P2**, and **P9** (three independent simulations were performed, for a total of 1.5 μs for each complex). The systems were built from the structure of the AM0627^E326A^–**P1**–Zn^2+^ complex as a template, upon reversing Ala326 to Glu326 (see the “Methods” section). The relative movement between the peptide and the enzyme was analyzed by monitoring selected intermolecular distances that are only formed when the peptide adopts reactive conformations. We selected two of the Zn^2+^ coordination distances (His324-N_ε_···Zn^2+^ and His329-N_ε_···Zn^2+^) and the interaction distances between Arg291 and the side chain of the peptide Gln (N_R291_···O_Q4_ and N_R291_···N_Q4_). The results showed that the naked peptide **P9** is highly dynamic and unstable, not being able to keep all these interactions at any time during the simulations, and frequently adopting conformations in which the peptide is almost detached from the enzyme (Fig. 4d). In contrast, **P2** and **P1** were stable for significant periods of time, both keeping these relevant interactions during the simulations  (Fig. 4c–f and Supplementary Figs. 5–8). These results agree with the experimental results reported above, showing that the enzyme preferentially recognizes **P1** and **P2** and easily binds **P1** in a suitable configuration for the catalytic reaction to take place.

### QM/MM metadynamics simulations of the reaction catalyzed by AM0627 suggest a water molecule acting as a nucleophile and a Glu residue as the catalytic base

**Fig. 5:**
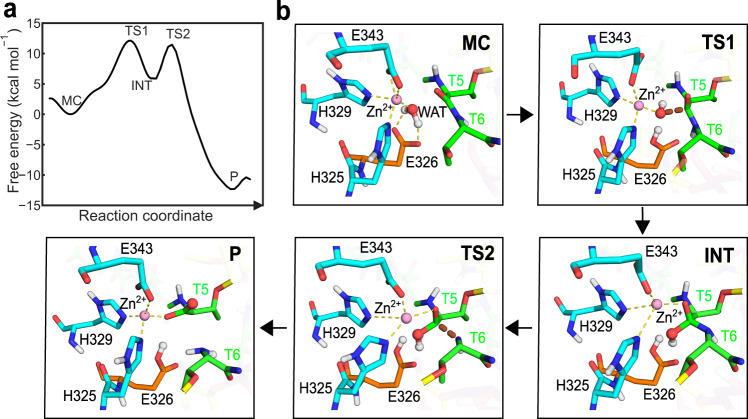
Reaction mechanism of AM0627 obtained from QM/MM metadynamics simulations. **a** Reaction free energy profile. **b** Representative structures of the Michaelis complex (MC), the transition state of the first reaction step (TS1), the reaction intermediate (INT), the transition state of the second step (TS2), and the reaction product (P) are depicted. The bold dashed lines (brown) represent bonds that are being formed or broken at the transition states. Broken yellow lines represent the coordination sphere of the Zn cation.

To address the catalytic mechanism of the enzyme, we performed QM/MM metadynamics simulations of the AM0627–**P1**–Zn^2+^ complex. Two distinct mechanisms have been proposed in the literature for Zn-metalloproteases, depending on whether the nucleophile residue (supposedly Glu326 in AM0627) directly attacks the carbonyl carbon atom of the scissile peptide bond (hereafter named as C*) or it does it indirectly, via a water molecule (Supplementary Fig. 9)^31,32^. The active site configuration of AM0627–**P1**–Zn^2+^ obtained from QM/MM MD simulations shows that the Zn^2+^ cation keeps the usual tetrahedral^33^ coordination and one oxygen atom of the Glu326 carboxylate group remains close to the C* atom (≈4 Å) (Supplementary Fig. 10a). This is a possible reactive configuration for direct nucleophilic attack of Glu326 on the peptide carbonyl. However, QM/MM metadynamics simulations of the chemical reactions considering direct attack from Glu326, using a collective variable corresponding to the Glu326–O···C* distance, resulted in a high energy barrier and the formation of an unstable complex (Supplementary Fig. 10b–d). This suggests that the nucleophilic attack of Glu326 is not direct but probably mediated by a water molecule. In fact, a water molecule was found to fit perfectly in the active site (Fig. 5b and Supplementary Fig. 11), being stable all along the QM/MM MD simulations. Such putative catalytic water is coordinated with the Zn^2+^ ion and forms a hydrogen bond with Glu326. The water molecule oxygen atom is at ≈3 Å from C*, being also well oriented for nucleophilic attack on the C* atom. To drive the reaction, we again used QM/MM metadynamics simulations of the nucleophilic attack, using the distance between the water oxygen and C* as a collective variable (Supplementary Fig. 11). The simulations led to the formation of a stable intermediate (INT, Fig. 5b) in which the C* atom is four-coordinated. The C*–N bond at INT is stretched with respect to its value at the Michaelis complex (from 1.39 Å at MC to 1.52 Å at INT) but not (yet) cleaved. During the reaction, a proton transfers from the attacking water molecule to Glu326, which thus acts as a general base. The free energy barrier of the reaction (12.1 kcal/mol, Fig. 5a) is indicative of a feasible reaction, in line with values previously reported for other Zn-dependent proteases^34–36^. A subsequent QM/MM metadynamics simulation was performed for the second reaction step of the enzymatic reaction, starting from the tetrahedral intermediate (INT), using the peptide bond distance (C*–N) as a collective variable (Supplementary Fig. 11). The simulations show that peptide bond cleavage is concomitant with the transfer of the C*–OH proton to the N atom. The whole process involves a much lower energy barrier (5.6 kcal/mol, Fig. 5a) than the first reaction step and leads to the complete cleavage of the C*–N bond (Fig. 5b). Therefore, AM0627 can effectively hydrolyze the T5–T6 peptide bond of **P1** in a two-step reaction, with the formation of a tetrahedral intermediate, via a nucleophilic attack by a water molecule and the assistance of Glu326 as a general base.

### Structural analysis of AM0627 and other mucinases can predict those displaying bis-*O*-glycan preferences

**Fig. 6:**
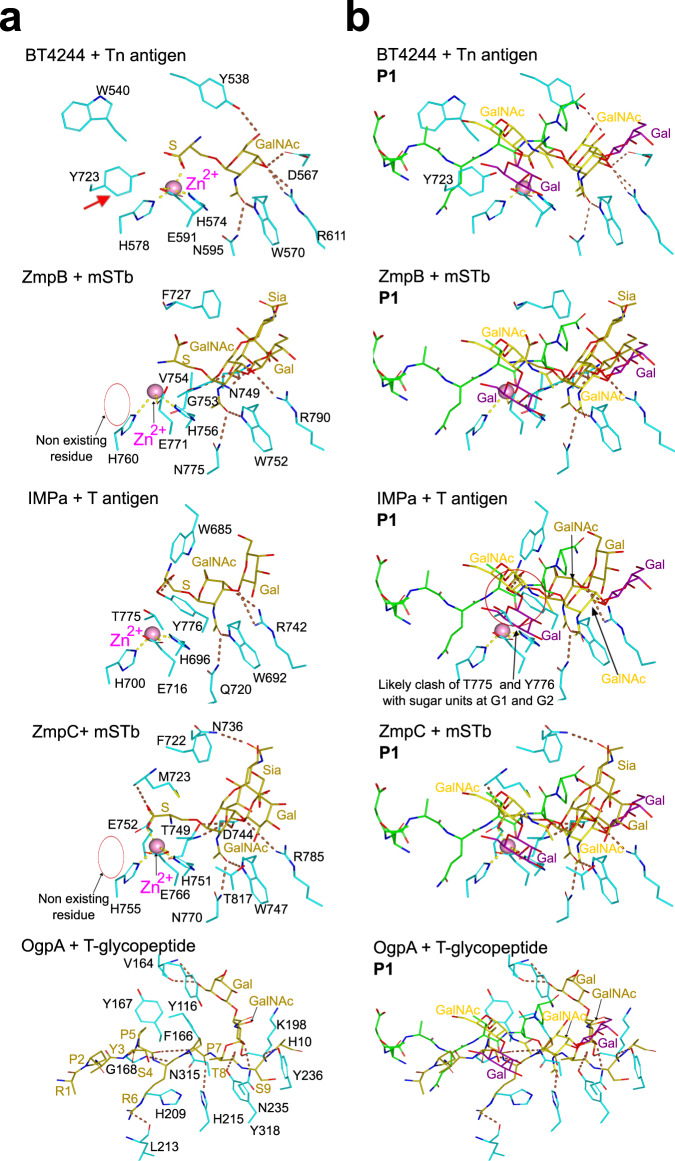
Analysis of BT4244, ZmpB, IMPa, ZmpC, OgpA, and AM0627 active sites. **a** Close-up view of the active sites of BT4244-Tn *O*-glycan, ZmpB,-mSTb, IMPa-T *O*-glycans, ZmpC-mSTb, and OgpA-T-glycopeptide complexes. Note that the zinc ion is not present in the structure of OgpA in complex with the T-glycopeptide because an inactive version of OgpA was used in which the His205, a residue that coordinates the Zn^2+^, and the catalytic Glu206 was mutated to Ala^20^. The ligands and the Zn^2+^ in these structures are shown as olive carbon atoms and as a pink sphere, respectively. The amino acids of the enzymes are shown as cyan carbon atoms. The dotted lines are displayed as brown and yellow for the hydrogen bonds and the interactions between the metal and the amino acids, respectively. **b** Active sites of BT4244-Tn *O*-glycan, ZmpB,-mSTb *O*-glycan, IMPa-T *O*-glycan, ZmpC-mSTb *O*-glycan, and OgpA-T-glycopeptide complexes showing the superimposed **P1** from AM0627^E326A^–**P1**–Zn^2+^ complex. **P1** is colored as in Figs. 2 and 3. The red arrow indicates the presence of the conserved Tyr723^BT4244^ with Tyr470^AM0627^, and the red circle indicates that the Tyr is not present in ZmpB/ZmpC and that a steric clash might occur between Thr775/Tyr776^IMPa^ and the sugar units at G1 and G2.

To infer why AM0627 recognizes clustered *O*-glycans and in turn cleaves glycopeptides containing bis-*O*-glycans, we analyzed the active sites of previously reported mucinases and the *O*-glycoprotease OgpA compared to that of AM0627. The structure of BT4244^28^ (PDB entry: 5KD8), ZmpB^28^/ZmpC^22^ (PDB entries: 5KDU/6XT1), IMPa^28^ (PDB entry: 5KDX), and OgpA^20^ (PDB entry: 6Z2P) were previously solved in complex with Tn, mSTb, and T *O*-glycans, and T-glycopeptide (i.e. a peptide containing a T *O*-glycan), respectively (Fig. 6a). Inspection of their active site reveals that the residues that are around the GalNAc moiety at the G1’ subunit (Trp321/Asn347/Arg362/Tyr288^AM0627^, Trp570/Asn595/Arg611/Tyr538^BT4244^, Trp752/Asn775/Arg790/Phe727^ZmpB^, Trp692/Gln720/Arg742/Trp685^IMPa^, and Trp747/Asn770/Arg785/Phe722^ZmpC^) and even their interactions with the GalNAc moiety are mostly conserved (Fig. 6a). Exceptions occur with Phe727^ZmpB^/Phe722^ZmpC^ that establish CH–π interactions with the Sia moiety of mSTb, and Trp685^IMPa^ that does not interact with any sugar moiety. Apart from these interactions at the G’ subunits, other interactions have been described thoroughly before^22,29^. Regarding the degree of conservation of the residues at the G subunits, only BT4244 shares the key tyrosine residue that was found to be crucial in AM0627 (Tyr470^AM0627^ and Tyr723^BT4244^). We showed above that Tyr470^AM0627^ interacts with both GalNAc and Gal located at the G1 and G2 subunits, respectively, and its mutation to Ala abolishes completely the AM0627 activity (Fig. 3b). Therefore, there are important structural similarities among these mucinases, indicating that some of them could behave similarly regarding *O*-glycan recognition.

To get more insights into the potential function of Tyr723 in BT4244, we superimposed the AM0627 crystal structure with those of BT4244, ZmpB, IMPa, and ZmpC (Fig. 6b; in this figure, only the **P1** from the AM0627^E326A^–**P1**–Zn^2+^ complex is shown for illustration purposes). As expected, the lowest root-mean-square deviation (RMSD) and the greater number of aligned residues were found between AM0627 and BT4244 (2.02 Å and 394 aligned residues, respectively) followed by ZmpB/ZmpC and AM0627 (2.25/2.26 Å and 351/342 aligned residues), and IMPa with AM0627 (2.99 Å and 342 aligned residues). A closer inspection of the active site of the complex between BT4244 and the Tn *O*-glycan and the superimposed **P1** (taken from the AM0627^E326A^–**P1**–Zn^2+^ complex) reveals that Tyr723 will likely recognize *O*-glycans located in the G subunits (Fig. 6b). This suggests that AM0627 and BT4244 should behave very similarly in terms of recognition towards bis-*O*-glycan and that BT4244 likely cleaves glycopeptides containing bis-*O*-glycans. In fact, it has been recently shown that BT4244 acts on glycopeptides with bis-*O*-glycans, in particular GS*T*A and VT*S*A motifs of the Tn-MUC1-TR reporter, while it is inactive in a single PDT*R *O*-glycosite^24,37^ (S* or T* denotes a GalNAc-glycosylated Ser and Thr, respectively). With respect to the other mucinases, while ZmpB and ZmpC do not have an aromatic residue close to the sugars at the G subunits, IMPa contains two threonines (Thr775 and Tyr776) that will likely clash with both the GalNAc and Gal at the G1 and G2 subunits, respectively. Therefore, IMPa does not have a suitable binding site to accommodate the sugar units located at the G subunits. Overall, the structural analysis shows that the absence of an aromatic residue suitably positioned to interact with the sugar units at the G subunits is the reason why ZmpB, ZmpC, and IMPa do not cleave on glycopeptides containing bis-*O*-glycans.

Finally, an inspection of the OgpA-active site shows that it is very different from that of AM0627, BT4244, ZmpB, IMPa, and ZmpC. This is exemplified by the large RMSD (4.50 Å) and the small number of aligned residues (only 111) between AM0627 and OgpA. In addition, not only the residues interacting with the sugar moieties of the T-glycopeptide are different (e.g. Val164, Phe166, Tyr236, and Lys198), but also the orientation of the T *O*-glycan in the T-glycopeptide with respect to the T *O*-glycan of **P1** that is located at the G’ subunits (Fig. 6a, b). Interestingly, although all mucinases analyzed here exhibit an aromatic residue in the vicinity of the GalNAc at the G1’ subunit (e.g. Tyr116^OgpA^, Tyr288^AM0627^, Tyr538^BT4244^, Phe727^ZmpB^, Trp685^IMPa^, Phe722^ZmpC^), this residue in OgpA (Tyr116) prefers to interact with the Gal at the G2’ subunit, while residues such as Phe727^ZmpB^/Phe722^ZmpC^ interact with the Sia moiety of mSTb. Therefore, there are enormous differences between OgpA and the other mucinases at the level of the active site and recognition of the *O*-glycans. In addition, the superposition of both structures suggests that some residues of OgpA, such as Asn315, will likely clash with the sugar moieties at the G subunits, explaining why OgpA is not able to cleave a peptide bond in a bis-*O*-glycan patch. At the level of the recognition of the substrate peptide sequence by OgpA, it is important to highlight that OgpA mostly recognizes the amino acid backbones of the T-glycopeptide, except for a hydrogen bond interaction between Arg6 side chain and Leu213 backbone (Fig. 6a), suggesting that OgpA, as a typical *O*-glycoprotease, might be less specific for the peptide sequences of glycoproteins substrates than AM0627. Yet, AM0627 is promiscuous towards different glycoprotein substrates, as shown very recently^25^.

### BT4244 preferably acts on glycopeptides with bis-Tn and bis-T *O*-glycans present in MUC1

**Fig. 7:**
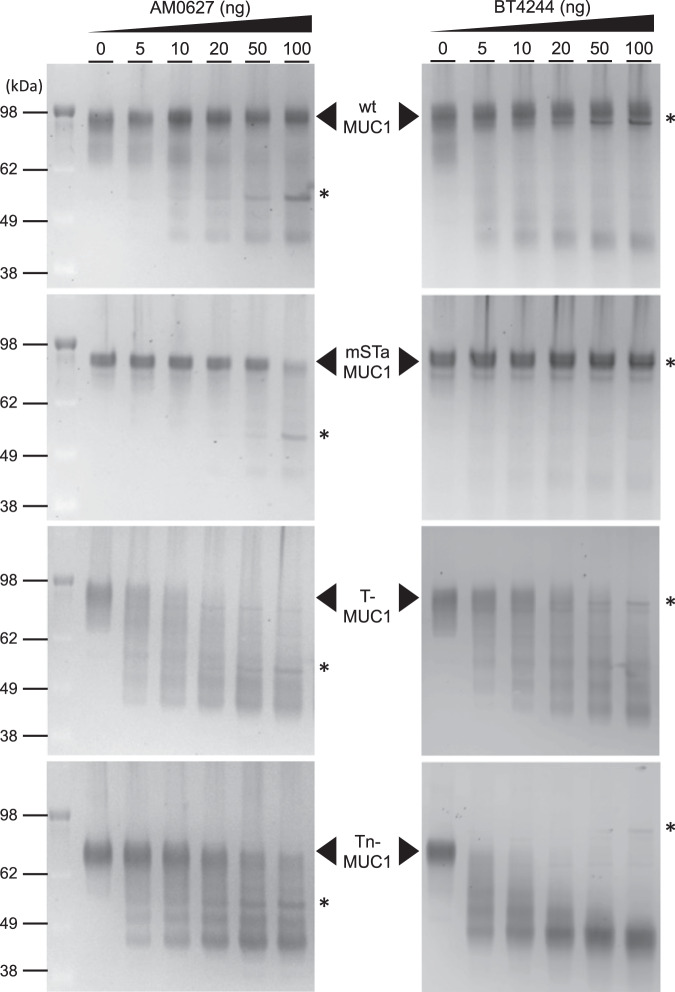
Analysis of AM0627 and BT4244 activity with isolated secreted MUC1-6.5xTRs reporters. SDS–PAGE analysis of wt AM0627^A21-E506^ and BT4244 (dose titration) digestion of secreted purified MUC1-6.5xTRs reporters produced in glycoengineered HEK293 cells (WT, mSTa, T, and Tn *O*-glycoforms). Purified reporters (0.5 µg) were incubated for 3 h at 37 °C with indicated enzyme and gels visualized with Krypton fluorescent protein stain. * indicates the bands corresponding to wt AM0627^A21-E506^ and BT4244. The experiments were performed in duplicate.

To get more insights into the activity of AM0627 and BT4244 on bis-*O*-glycans, we compared the activity of BT4244 and wt AM0627 towards different *O*-glycan forms using a recombinant mucin reporter *O*-glycoprotein substrate, which contains 6.5 TRs of the 20 amino acid human MUC1 TR sequence (GV**TS**APD**T**RPAPG**ST**APPAH) with five *O*-glycosites. Note that the MUC1 TR contains two bis-*O*-glycosites and one isolated glycosite. We previously showed that all five *O*-glycosites are fully *O*-glycosylated when expressed in glycoengineered HEK293 cells and that BT4244 efficiently cleaves the Tn glycoform of this MUC1 reporter with predominant cleavage in between the bis-*O*-glycan at the VTSA and GSTA motifs^24,37^. We tested the wt glycoform of the MUC1 TR reporter (containing a mixture of mono and disialylated core 1 and core 2 structures), and three engineered more homogeneous glycoforms with mSTa, T, and Tn *O*-glycans. AM0627 was previously suggested to cleave bis-mSTa *O*-glycans^19^. However, here we found that neither AM0627 nor BT4244 efficiently cleaved the sialylated MUC1 reporters (wt MUC1 and mSTa-MUC1), although slight degradation of the mSTa glycoform by AM0627 at the highest concentration (1:5 enzyme/substrate ratio) was apparent. In contrast, both mucinases efficiently cleaved the T and Tn-MUC1 reporters (cleavage observed from 1:100 enzyme/substrate ratio) (Fig. 7), which is in agreement with the cleavage studies using the **P** glycopeptides (Fig. 4b). AM0627 exhibited a preference for the T glycoform compared to Tn, while BT4244 revealed the inverse pattern with a preference for the Tn glycoform. To rationalize this data, we performed MD simulations on the complex of BT4244 with a MUC1 glycopeptide (AHGV**TS**A) containing a bis-T and a bis-Tn *O*-glycan. The results show that the hydrogen bonds established between BT4244 and the bis-Tn GalNAc at the G1’ subunit are more stable during the simulation than the hydrogen bonds between BT4244 and the bis-T GalNAc at the G1’ subunit, which might explain why BT4244 slightly prefers to act on glycopeptides containing bis-Tn over bis-T patches (Supplementary Fig. 12).

To further explain why both mucinases did not act on glycopeptides containing sialic acids (bis-mSTa), we inspected the active site of the structure of AM0627 in complex with **P1**. Our analysis reveals that the Sia bound to the OH3 of Gal at the G2 subunit would likely clash with residues such as Lys387, Asp388, and Val389. Note that Val389 is at 3.38 Å from the Gal OH3 (Supplementary Fig. 13a). Regarding the other Gal at the G2’ subunit, it is likely that the Sia moiety would be repulsed by the negatively AM0627 surface close to the Gal moiety (Supplementary Fig. 13b). Finally, we also addressed why the STn glycoform blocks peptide cleavage, as reported for the StcE mucinase^5^ and other mucinases^19^. A closer look at the AM0627-active site reveals that α2-6-linked Sia residues on the GalNAc moieties will likely clash with Trp149, Tyr288, and Asp318. Note that the OH6 of GalNAc at the G1 subunit is at 5.25 Å of Trp149, and the OH6 of GalNAc at the G1’ subunit is at 3.38 Å of Tyr288, and 2.47 Å of Asp318 (Supplementary Fig. 13a). Overall, our experimental results clearly demonstrate that BT4244 has the same preferences for bis-*O*-glycans as those of AM0627, although with somewhat different cleavage rates. In addition, our MD simulations suggest that steric factors are the reason for the observation that these two mucinases do not efficiently act on glycoproteins with *O*-glycans capped by Sia as demonstrated in our experiments with the MUC1 mucin reporters (Fig. 7). Recent studies have suggested that AM0627 can cleave native MUC2 isolated from Caco-2 cells although with increased efficiency after desialylation^25^, and analysis of select cleaved fragments have shown mST *O*-glycans at the cleavage site^19,25^. However, these studies were performed with extremely high enzyme-to-substrate ratio and extended digestion times (24 h). In addition, native isolated mucins are highly heterogeneous substrates for which it is not possible to efficiently monitor the degradation (e.g. cleavage at one or few sites versus cleavage at all potential bis-*O*-glycan sites). The present data with well-defined *O*-glycosylated substrates and normalized enzyme to substrate ratios clearly support the structural data that sialic acids and more elaborate *O*-glycan structures are much poorer substrates.

## Discussion

Degradation of mucins is important for the normal process of renewal and clearance of mucins in the mucus layers lining mucosal surfaces and essential for pathogenic bacteria like enterohemorrhagic *Escherichia coli* (EHEC) relying on the penetration of the protective mucus layers to reach the underlying epithelium^5,23,38,39^. While several mucinases have been reported and characterized so far, only two of these, StcE and ZmpC, derived from pathogenic bacteria, appear to be able to cleave mucins covered by complex elaborated *O*-glycan structures (Fig. 1b). Such mucinases capable of cleaving nascent mature mucins with complex sialylated and fucosylated *O*-glycans do not depend on prior trimming of the *O*-glycans by other glycoside hydrolases normally produced by the microbiota as part of the process of degrading mucins for nutrient sourcing^40,41^. In contrast, mucinases such as AM0627 and BT4244 that only cleave mucins with short truncated *O*-glycans are likely dependent on prior trimming of *O*-glycans down to Tn or T by bacterial glycosidases produced by commensal and symbiotic bacteria. Although further insights are clearly needed, we envision that mucinases like AM0627 and BT4244 may serve in the last steps of degradation of the mucus layer during its continuous renewal to ensure homeostasis of the process^1^. *Akkermansia muciniphila* is a gut commensal that relies on mucins as the main source of carbon and nitrogen, and this bacteria highly expresses AM0627 as well as multiple glycosidases and putative sulfatases in response to mucins^42^.

Mucinases and *O*-glycoproteases characterized thus far appear to rely on a single *O*-glycan adjacent to the peptide cleavage site, and here we unveiled molecular mechanisms by which the mucinases AM0627 and BT4244 instead exhibit preference for cleavage in between two or more *O*-glycans. Clustered *O*-glycan patches are particularly found in mucins and mucin-like domains, with MUC2 and MUC5AC being among the mucins with the highest density of *O*-glycans including stretches of 3–6 adjacent *O*-glycans. While the sequence and spacing of *O*-glycans in mucins may not be conserved, the density of *O*-glycans does appear to be conserved. On the contrary, the distribution of bis *O*-glycans is found among most mucin TRs and many O-glycoproteins^3^. Our structure of an inactive form of AM0627 with **P1** shed light into how this mucinase recognizes bis-*O*-glycans and achieves catalysis. AM0627 clearly prefers substrates containing bis-T *O*-glycans and to lesser degree bis-Tn with a critical need for a GalNAc moiety at the G1 subunit for catalysis. Based on the AM0627 structure and its comparison with other mucinases and *O*-glycoproteases, we also inferred that a Tyr residue (Tyr470) is key to explain the recognition of bis-*O*-glycans, a feature shared in the BT4244 mucinase with similar substrate preference. The recently reported crystal structure of the Zn^+2^-bound AM0627 did not reveal the molecular basis for the bis-*O*-glycan substrate preference^25^, likely due to misplacement of the mono-T *O*-glycan at S1 with sugar moieties located at G1 and G2 subunits. This study also concluded that the *O*-glycans were in close contact with Trp149 and Phe290 residues^25^, which clearly is not the case in our crystal structure. Misplacement of the docked glycopeptide substrate may also have been the reason for missing the key function of Tyr470 in recognition of the mono-T *O*-glycan at S1 in driving the preference for bis-*O*-glycan substrate sites. Interestingly, this recent study included activity analyses of enzyme mutants (mutations at residues Trp149, Tyr287, and Phe290) using different glycoprotein substrates that showed the importance of these residues in the recognition of the peptide backbone of glycoprotein substrates^25^, which further support our interpretation of the AM0627 crystal structure presented here. Overall, this demonstrates the importance of obtaining experimental structures of protein–ligand complexes for reliable information on protein recognition and mechanisms that may be further complemented with computational studies.

The finding that the AM0627 and BT4244 mucinases does not use MUC1 TR reporter substrates with *O*-glycans capped by Sia is supported by our structural analysis. Shon et al. ^25^ previously inferred that AM0627 cleaved glycoproteins with Sia moieties. However, this interpretation was based on enzyme assays with highly heterogenous glycoprotein substrates and excessive enzyme ratios and incubation times. The study presented here, using mucin reporter substrates with defined *O*-glycan structures, clearly confirms that AM0627 and BT4244 have strong specificities for bis-*O*-glycans with either T or Tn glycoform preferences, and are expected to cleave more widely when substrates are presented with unsialylated T and Tn *O*-glycans.

Zinc metallopeptidases follow two distinct mechanisms for the cleavage of peptide sequences depending on whether the nucleophile is a water molecule^43^ or a Glu residue^44^. Based on our QM/MM metadynamics simulations, we found that AM0627, and likely other mucinases, follow a two-step mechanism in which a nucleophilic water molecule is firstly activated by a Glu residue acting as the catalytic base (Glu326 in AM0626). The first and most important step of catalysis leads to the formation of an intermediate with tetrahedral coordination of the carbon atom of the T5–T6 peptide bond, which is effectively cleaved in the second catalytic step, assisted by the transfer of a proton from T5 to T6. A water molecule that establishes a hydrogen bond with the general base residue (Glu326) is well poised for nucleophilic attack on the carbonyl group of the scissile peptide bond during the first reaction step. Interestingly, a water molecule was also observed near the GalNAc acetamide NH at G1 subunit in the X-ray structure of AM0627^E326A^ in complex with **P1**, thus we speculate that this water molecule is held by Glu326 in the complex of the WT enzyme and acts as the nucleophile. The kinetics data with the inactive **P7**, which contains a galactosamine at G1, thus lacking the acetamido group, suggested that this group is crucial for catalysis. It is possible that the acetamido group has a role in sequestering the catalytic water molecule through the NH substituent. In addition, the absence of the acetyl group in the galactosamine moiety might lead to the formation of a protonated amine group that might not be efficient in trapping the catalytic water molecule, and/or influence the position of the sugar moiety bound to AM0627. Both scenarios would certainly affect negatively the catalytic properties of AM0627.

In conclusion, we provide structural and mechanistic insights into a mucinase that recognizes bis-*O*-glycans, which have led to the identification of another mucinase, BT4244, with the same requirement for bis-*O*-glycans, albeit with a different preference for *O*-glycan structures. We identified a key conserved Tyr residue in AM0627 and BT4244 positioned close to the substrate G subunits responsible for the preference for bis-*O*-glycans. The expanding repertoire of mucinases provides new tools to break a barrier in studying mucins and *O*-glycoproteins with dense *O*-glycodomains that cannot be digested by traditional proteases. Deeper knowledge of the substrate specificities of these mucinases, both with respect to peptide backbone and *O*-glycan positions and structures, will aid in the design of digestion strategies for select glycoprotein substrates.

## Methods

### Protein expression and purification

The DNA sequence encoding amino acid residues 21–506 of the AM0627 (Amuc_0627) was codon optimized and synthesized by GenScript (USA) for expression in *E. coli*. At the 5′-end, the construct also contained a sequence encoding a 12xHis tag and a Tobacco Etch Virus (TEV) cleavage site. The DNA, containing at the 5′-end a recognition sequence for *EcoR*I, and at the 3′ end a stop codon and a recognition sequence for *Sal*I, was cloned into pMALC2x, rendering the vector pMALC2x-12Hist-TEV-*AM0627*^*21–506*^. In the vector, the TEV cleavage site is located between the maltose binding protein (MBP)-12Hist and the protein of interest. All mutants in AM0627 were generated following a standard site-directed mutagenesis protocol by GenScript (quick change) using the vector pMALC2x-12Hist-TEV-*AM0627*^*21–506*^. The vector pMALC2x-12Hist-TEV-*AM0627*^*71–506*^-E326A and pMALC2x-12Hist-TEV-*AM0627*^*71–506*^ were also generated by GenScript and by using as a template the *AM0627*^*21–506*^-E326A construct from pMALC2x-12Hist-TEV-*AM0627*^*21–506*^-E326A and the *AM0627*^*71–506*^-E326A from pMALC2x-12Hist-TEV-*AM0627*^*71–506*^-E326A, respectively.

Each plasmid was transformed into *E. coli* BL21(DE3) and grown in 2XTY medium (1.6% (w/v) tryptone, 1% (w/v) yeast extract powder and 0.5% (w/v) NaCl), containing 100 μg/ml of ampicillin at 37 °C. When the O.D at 600 nm reached ~0.6 to 0.8, the culture was induced with 1 mM isopropyl 1-thio-ß-d-galactopyranoside (IPTG) at 18 °C. After 16 h incubation, the cells were harvested by centrifugation at 17,700 × *g* at 4 °C for 10 min. Cells were lysed using buffer A (25 mM Tris pH 8, 500 mM NaCl, 10 mM imidazole) and loaded into a HisTrap Column (GE Healthcare). Proteins were eluted with an imidazole gradient from 10 mM up to 500 mM and then the buffer was exchanged to buffer B (25 mM Tris pH 8, 150 mM NaCl) using a HiPrep 26/10 Desalting Column (GE Healthcare). Thereafter, the TEV recognition site was cleaved using TEV protease.

TEV protease and MBP-12Hist were later removed from the solution using a His-Trap Column (GE Healthcare), and isolated proteins were then loaded into a HiLoad 26/60 Superdex 75 Colum (GE Healthcare), previously equilibrated with buffer B. The proteins were concentrated using Amicon Ultra-15 mL and quantification was carried out by absorbance at 280 nm using their theoretical extinction coefficient (*ε*_280nm_ for the wt AM0627^A21-E506^ and mutants = ~78730–78980 M^−1^ cm^−1^, and *ε*_280nm_ for wt AM0627^A71-E506^ and AM0627^E326A^ mutant = ~77240 M^−1^ cm^−1^).

Recombinant BT4244 enzyme was produced in *E. coli* and purified as reported previously^24,37^. Briefly, the recombinant BT4244 (residues 35–857) was gene synthesized with a codon-optimized sequence (Twist bioscience, USA) and cloned in a pET28-based vector. The plasmid was transformed in T7 Express (NEB) bacterial strains, grown at 37 °C for 2 h, induced with 1 mM IPTG, and cultured at 16 °C overnight. Proteins were purified by nickel-nitrilotriacetic acid (Ni-NTA) chromatography and followed by gel-filtration chromatography with a Superdex 200 16/60 column. The fraction containing the enzyme were pooled and dialyzed in PBS.

### Crystallization and data collection

AM0627^E326A^ was concentrated to 15 mg/ml and co-crystallized with 0.5 mM ZnCl_2_ and 5 mM **P1**. Appropriate size of crystals appeared at 0.07 M Monosaccharides, 0.1 M Buffer system 1 pH 6.5 and 30% precipitant mix 2 (Molecular Dimensions). The crystals were cryoprotected in mother liquor containing 25% glycerol and flash frozen in liquid nitrogen.

The data were collected in the beamline BL13 XALOC of ALBA at a wavelength of 0.97 and 1.28 Å and a temperature of 100 K. Data were processed and scaled using XDS^45^ and CCP4^46^ software packages. Single anomalous diffraction (SAD) using SHELXD^26^ was applied to the crystal collected at a wavelength of 1.28 Å, allowing us to find two zinc sites using the anomalous signal present until 1.9 Å resolution, one for each monomer, whose correlation coefficient was 43.0% for all data and 29.2% for weak reflections. Subsequently, SHELXE^26^ was used to distinguish correct handedness by density modification and to reveal the protein atoms using polyalanine tracing with helical and strand seeds. From this solution and with 3 autotracing cycles with 10 density modification cycles, SHELXE^26^ distinguished correct handedness and was able to trace 845 residues divided into 10 chains (corresponding to the two molecules in the AU), which are calculated in a correlation coefficient of 41.85%. Then, we solved the crystal structure of the AM0627^E326A^–**P1**–Zn^2+^ complex by molecular replacement with Phaser^27^ using the model from SHELXE. Initial phases were further improved by cycles of manual model building in Coot^47^ and refinement with REFMAC5^46^. Further rounds of Coot and refinement with REFMAC5 were performed to obtain the final structure. The final model was validated with PROCHECK;^46^ model statistics are given in Supplementary Table 1. The AU of the P2_1_2_1_2_1_ crystal contained two molecules of AM0627^E326A^. The Ramachandran plot for the AM0627^E326A^ shows that 90.6%, 8.6%, 0.3%, and 0.5% of the amino acids are in the most favored, allowed, generously allowed, and disallowed regions, respectively.

### Molecular dynamics (MD) simulations in explicit water

We used our crystal structure of AM0627^E326A^ and that of BT4224 (PDB entry: 5KD8^29^) as the starting structures for all simulations reported in this work. The mutation of the catalytic residue (E326A) was mutated back to Chimera^48^. The protonation states of His were chosen based on the hydrogen bond network and metal coordination manually check with Chimera. The Zn^2+^-coordinating His325 and His329 residues were inserted as N_δ_-protonated while the rest histidine residues were inserted as N_ε_-protonated. The simulations were performed at pH 7, thus all Asp and Glu residues were negatively charged while all Arg and Lys residues were positively charged. The complex system was placed in the center of a cubic box 98 × 98 × 98 Å^3^ with a distance of at least 10 Å between the surface of the solute and the edge of the box. The box was then solvated with TIP3P water molecules, and counterions were added to neutralize the system. The protein was described using the Amber ff14SB force field^49^, while the GLYCAM06 force field^50^ was used to describe the carbohydrate molecules. The LEaP module of AMBER20 was used to generate the topology and coordinate files for the classical MD simulations, which were carried out using the CUDA version of the PMEMD module^51^ of the AMBER20 simulation package. The solvated system was first subjected to 5000 steps steepest descent minimization, followed by 5000 steps conjugate gradient minimization with positional restraints on all heavy atoms of the solute, using a 50 kcal mol^−1^ Å^−2^ harmonic potential. The minimized system was then heated up to 300 K using the Berendsen thermostat, with a time constant of 1 ps for the coupling, and 50 kcal mol^−1^ Å^−2^ positional restraints applied over three 500 ps steps of the heating process. The positional restraints were then gradually decreased to 5 kcal mol^−1^ Å^−2^ over four 500 ps steps of NPT equilibration, using the Berendsen thermostat and barostat to keep the system at 300 K and 1 atm. For the production runs, each system was subjected to either 200 or 400 ns of sampling in an NPT ensemble at constant temperature (300 K) and constant pressure (1 atm), controlled by the Langevin thermostat, with a collision frequency of 2.0 ps^−1^, and the Berendsen barostat with a coupling constant of 1.0 ps. The SHAKE algorithm was applied to constrain all bonds involving hydrogen atoms. A cut-off of 10 Å was applied to all nonbonded interactions, with the long-range electrostatic interactions being treated with the particle mesh Ewald (PME) approach. A time step of 2 fs was used for all the classical simulations, and coordinates were saved from the simulation every 10 ps. Three independent runs were performed.

### QM/MM metadynamics

One representative snapshot extracted from the classical MD trajectory was used for the subsequent QM/MM MD simulations, which combines Born–Oppenheimer MD simulation, based on density functional theory (DFT), with force-field MD methodology. The QM region consists of the Zn^2+^ ion and its coordinated residues (His325, His326, and Glu343) as well as parts of the substrate peptide (Thr5, Thr6, and Pro7) and the catalytic water molecule and Glu326, resulting in a total number of 76 QM atoms (including 7 capping hydrogens), as shown in Supplementary Fig. 11. The dangling bonds between the QM and MM region were capped with hydrogen atoms. The QM region was enclosed in an isolated supercell of size 20.0 × 20.0 × 20.0 Å^3^. All QM/MM MD and metadynamics simulations were performed using CP2K v7.1 interfaced with PLUMED v2.5^52,53^, combining the QM program QUICKSTEP and the MM driver FIST. In this code, a real space multigrid technique is used to compute the electrostatic coupling between the QM and MM^54^ region. The QM region was treated at the DFT (BLYP) level, employing the dual basis set of Gaussian and plane-waves (GPW) formalism, whereas the remaining part of the system was modeled at the classical level using the same parameters as in the classical MD simulations. The Gaussian triple-ζ valence polarized (TZV2P) basis set was used to expand the wave function, while the auxiliary plane-wave basis set with a density cut-off of 350 Ry and GTH pseudopotentials^55^ was utilized to converge the electron density. All QM/MM MD simulations were performed under the NVT ensemble using a coupling constant of 10 fs and an integration time step of 1.0 fs. First, the system was equilibrated without any constraint for 10.0 ps. Then, the metadynamics^53^ method was used to explore the free energy profile for each reaction step. The distance between water oxygen and carbonyl carbon of Thr5 (C*) was used as a collective variable (CV1) for the first reaction step, while the distance between carbonyl carbon of Thr5 and amide nitrogen of Thr6 was taken as a collective variable for the second reaction step (Supplementary Fig. 11). The proton transfers happened spontaneously during the metadynamics simulations, so no CV was needed for activating them. The Gaussian height was set to 1.0 kcal/mol, which was reset to 0.1 kcal/mol when it was about to cross the transition state, and the time deposition interval between two consecutive Gaussians was set to 25 fs. Gaussian widths were tuned according to the oscillations of each collective variable (0.2 Å for both CV1 and CV2). Recrossing over the TS^52^ was observed in the first reaction step, but not the second step. Here, the proton transferred from Thr5 to the amide group of Thr6 spontaneously but did not return to the intermediate state. For this reason, the free energy of the P state is likely to be overestimated, while the reaction mechanism is not affected. The MD trajectories obtained in the simulation were analyzed by VMD and PyMOL (PyMOL 2.4.2), and distance calculation and clustering analysis were done by CPPTRAJ^56^ from Amber 20. Plots were made with Matplotlib, while figures of structures were rendered with Chimera^48^ and PyMOL, plots and figures were combined with Inkscape v0.92.3.

### Solid-phase (glyco)peptide synthesis (SPPS)

(Glyco)peptides were synthesized by stepwise microwave-assisted solid-phase synthesis on a Liberty Blue synthesizer using the Fmoc strategy on Rink Amide MBHA resin (0.1 mmol). Fmoc-Thr[GalNAc(Ac)_3_-α-D]-OH (2.0 equiv) or Fmoc-Thr(GalN_3_(Ac)_3_-α-D]-OH (2.0 equiv) was synthesized as described in the literature^57^ and manually coupled using HBTU [(2(1H-benzotriazol-1-yl)-1,1,3,3-tetramethyluronium hexafluorophosphate], while all other Fmoc amino acids (5.0 equiv.) were automatically coupled using oxyma pure/DIC (N,N’-diisopropylcarbodiimide). The *O*-acetyl groups of GalNAc moiety were removed in a mixture of NH_2_NH_2_/MeOH (7:3). In the case of peptides **P7** and **P8**, the azido group was transformed into the corresponding amino group by standard Pd/C hydrogenation. (Glyco)peptides were then released from the resin, and all acid-sensitive side-chain protecting groups were simultaneously removed using TFA 95%, TIS (triisopropylsilane) 2.5% and H_2_O 2.5%, followed by precipitation with cold diethyl ether. The crude products were purified by HPLC on a Phenomenex Luna C18(2) column (10 μm, 250 mm × 21.2 mm) and a dual absorbance detector, with a flow rate of 10 mL/min.

### Glycopeptide preparation

All the glycopeptides used in this work were dissolved at 100 mM in buffer 25 mM Tris pH 7.5. The pH of each solution was measured with pH strips and when needed adjusted to pH 7–8 through the addition of 0.1–5 μL of 2 M NaOH.

### Synthesis of glycopeptides containing the T *O*-glycan

The glycopeptide **P1** was incubated overnight at 37 °C at 23 mM with 51 μM *D. melanogaster* C1GalT1^58^, 200 μM MnCl_2_ and 97 mM UDP-Gal in buffer B in a final volume of 180 μL. To generate **P3** and **P4**, we used similar conditions to the described above but used the substrates **P5** and **P6**, respectively. Purification of the new glycopeptides was performed as described above.

### In vitro enzyme cleavage of glycopeptides

In vitro glycopeptidase cleavage activity was measured by MALDI-TOF MS semi-quantitatively. The reaction of the wild-type (wt) AM0627^A21-E506^ and all mutants were performed by adding 0.4 μM of the wt AM0627^A21-E506^ and mutants with 57 μM of **P1** and incubated at 37 °C in 50 mM ammonium bicarbonate buffer (pH 8.0). The experiments with **P1**–**P9** were performed by adding 0.4 μM of the wt AM0627^A21-E506^ with 500 μM of the (glyco)peptides in 50 mM ammonium bicarbonate buffer and incubated at 37 °C. The reaction mixtures were taken at the indicated time points and product development was detected by MALDI-TOF MS.

### Proteolytic Cleavage Assay with MUC1-6.5xTRs reporter

The design and construction of the MUC1-6.5xTRs reporters were previously reported^5^. Glycoengineered HEK293 cell lines (HEK293 cells were originally purchased from GIBCO) with *O*-glycan designs for Tn (knockout (KO) *C1GALT1*), core 1 (KO *GCNT1*, *ST3GAL1/2*, *ST6GALNAC2/3/4*), mono-sialylT (mSTa) (KO *GCNT1*, *ST6GALNACT2/3/4*) and wildtype HEK293^WT^ were used for the stable expression of MUC1 TR reporter and are available as part of the cell-based glycan array resource^59^. All isogenic HEK293 cells stably expressing MUC1-TR reporters were seeded at a density of 0.25 × 10^6^ cells/ml and cultured for 5 days on an orbital shaker in F17 medium (Gibco) supplemented with 0.1 Kolliphor P188 (Sigma-Aldrich) and 2% Glutamax. Culture media were purified by Ni-NTA affinity (Qiagen) chromatography (pre-equilibration with 25 mM sodium phosphate, 0.5 M NaCl, 10 mM imidazole pH 7.4, and eluted with the addition of 200 mM imidazole). Purified reporters were further desalted followed by buffer exchange to MiliQ using Zeba spin columns (Thermo Fisher Scientific) and quantified using a Pierce™ BCA Protein Assay Kit (Thermo Fisher Scientific). Proteolytic cleavage assays with purified glycoengineered MUC1 reporters (500 ng) were performed by incubating serial dilutions of the wt AM0627^A21-E506^, AM0627^P71-E507^, or BT4244 for 2 h at 37 °C in 50 mM ammonium bicarbonate buffer pH 8.0. Reactions were terminated by heat inactivation at 95 °C for 5 min. Samples were run on NuPAGE Novex gels (Bis–Tris 4–12%) at 200 V for 45 min followed by staining with Krypton Fluorescent Protein Stain (Thermo Fischer Scientific) and imaged with an ImageQuant LAS4000 system (GE Healthcare).

### Reporting summary

Further information on research design is available in the Nature Research Reporting Summary linked to this article.

## Supplementary information

Supplementary Information

Reporting Summary

## Data Availability

The crystal structure of the AM0627^E326A^–**P1**–Zn^2+^ complex was deposited at the RCSB PDB with accession code 7YX8. Previously published PDB structures used in this study are available under the accession codes 5KD8, 5KDU, 6XT1, 5KDX, 6Z2P and 7SCI. The trajectory files of the classical MD simulation and QM/MM metadynamics simulations have been deposited to Zenodo at 10.5281/zenodo.6521230. Other data are available from the corresponding author upon request. Source data are provided with this paper.

## Source data

Source Data
