# Supplementary material for: Structural and mechanistic insights into the cleavage of clustered O-glycan patches-containing glycoproteins by mucinases of the human gut
Source: Nat Commun. 2022 Jul 26;13:4324. doi: 10.1038/s41467-022-32021-9 (PMC9325726; doi:10.1038/s41467-022-32021-9)
Supplement: Supplementary file 1 — Supplementary Information [file 41467_2022_32021_MOESM1_ESM.pdf]

SUPPLEMENTARY INFORMATION

Structural insights into the cleavage of clustered *O*-glycan patches-containing glycoproteins by mucinases of the human gut

Víctor Taleb[1^], Qinghua Liao[2^], Yoshiki Narimatsu[3^], Ana García-García[1^], Ismael Compañón[4^], Rafael Junqueira Borges[5], Andrés Manuel González-Ramírez[1], Francisco Corzana[4], Henrik Clausen[3], Carme Rovira[2,6]* and Ramon Hurtado-Guerrero[1,3,7]*

[1] Institute of Biocomputation and Physics of Complex Systems, University of Zaragoza, Mariano Esquillor s/n, Campus Rio Ebro, Edificio I+D, Zaragoza, Spain.

[2] Departament de Química Inorgánica i Orgánica (Secció de Química Orgánica) and Institut de Química Teorica i Computacional (IQTCUB), Universitat de Barcelona, 08028 Barcelona, Spain

[3] Copenhagen Center for Glycomics, Department of Cellular and Molecular Medicine, University of Copenhagen, Copenhagen, Denmark.

[4] Departamento de Química, Universidad de La Rioja, Centro de Investigación en Síntesis Química, E-26006 Logroño, Spain.

[5] Departamento de Física e Biofísica, Instituto de Biociências, Universidade Estadual Paulista (UNESP), Botucatu, Brazil.

[6] Institució Catalana de Recerca i Estudis Avancats (ICREA), 08010 Barcelona, Spain.

[7] Fundación ARAID, 50018, Zaragoza, Spain.

^Víctor Taleb, Qinghua Liao, Yoshiki Narimatsu, Ana García-García and Ismael Compañón contributed equally to this work.

contributed equally to this work.

* To whom correspondence should be addressed: rhurtado@bifi.es and c.rovira@ub.edu

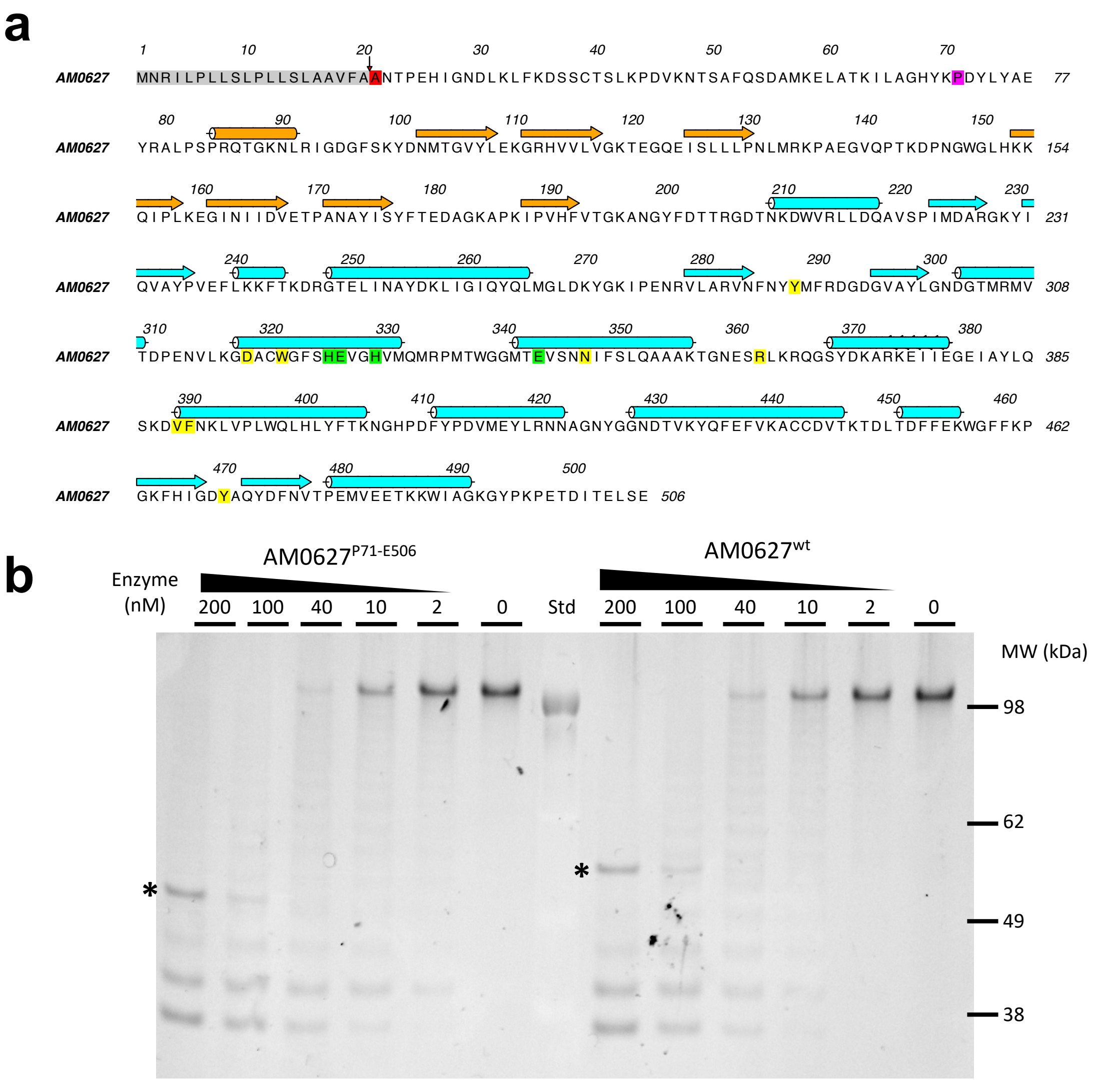

Supplementary Figure 1. a, Sequence of the AM0627**.** Shown above the sequence, in orange (IgG fold domain) and cyan (catalytic domain), are the secondary structure elements (α-helices and β-strands) based on the AM0627 structure. The residues His325, Glu326 and His329, forming the HEXXH motif, together with Glu343 are highlighted in green. Residues mutated in this work are highlighted in yellow. The signal sequence is predicted to encompass residues 1 to 20 by SignalP (residues highlighted in grey). The constructs used in this work start at Ala21 (red) and Pro71 (magenta; crystallizable construct), and finish in Glu506. An arrow indicates the predicted signal protein cleavage point. **b**, SDS-PAGE analysis of the activity of wt AM0627$^{A21\text{-}E506}$ and the truncated

AM0627$^{P71\text{-}E506}$ with an artificial Tn bis-*O*-glycan reporter. The artificial reporter contains eighteen 12-mer repeats with a bis-*O*-glycosite $(AEAAATTPAPAK)_{n=18}$ and was expressed in HEK293 cells with KO of *C1GALT1* for the Tn *O*-glycoform. The purified Tn bis-*O*-glycan reporter (0.5 µg) was incubated for 2 h at 37°C with the enzymes (0-200 nM). Gels were visualized with Krypton fluorescent protein stain. * indicates the bands corresponding to AM0627$^{P71\text{-}E506}$ and wt AM0627$^{A21\text{-}E506}$. The middle lane marked as std contains the protein molecular weight markers (note that the 98 kDa protein is the best visualized protein molecular weight marker of all due that Krypton does not stain the rest of the protein markers well). In addition, the MW for each protein is indicated on the right side. The experiments were performed in duplicate (n = 2).

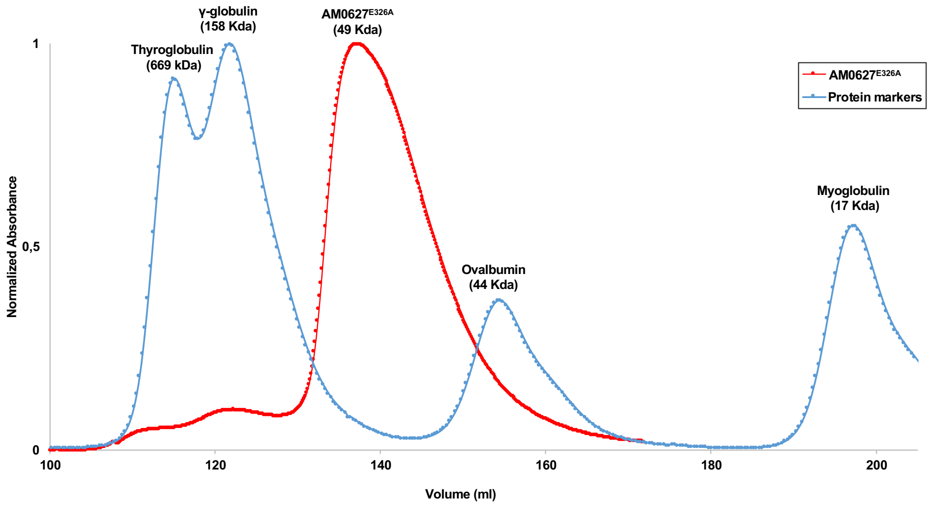

Supplementary Figure 2. Analysis of AM0627^{E326A} by size exclusion chromatography. The chromatogram shows several protein markers with their molecular weights and the monomeric form for AM0627^{E326A}.

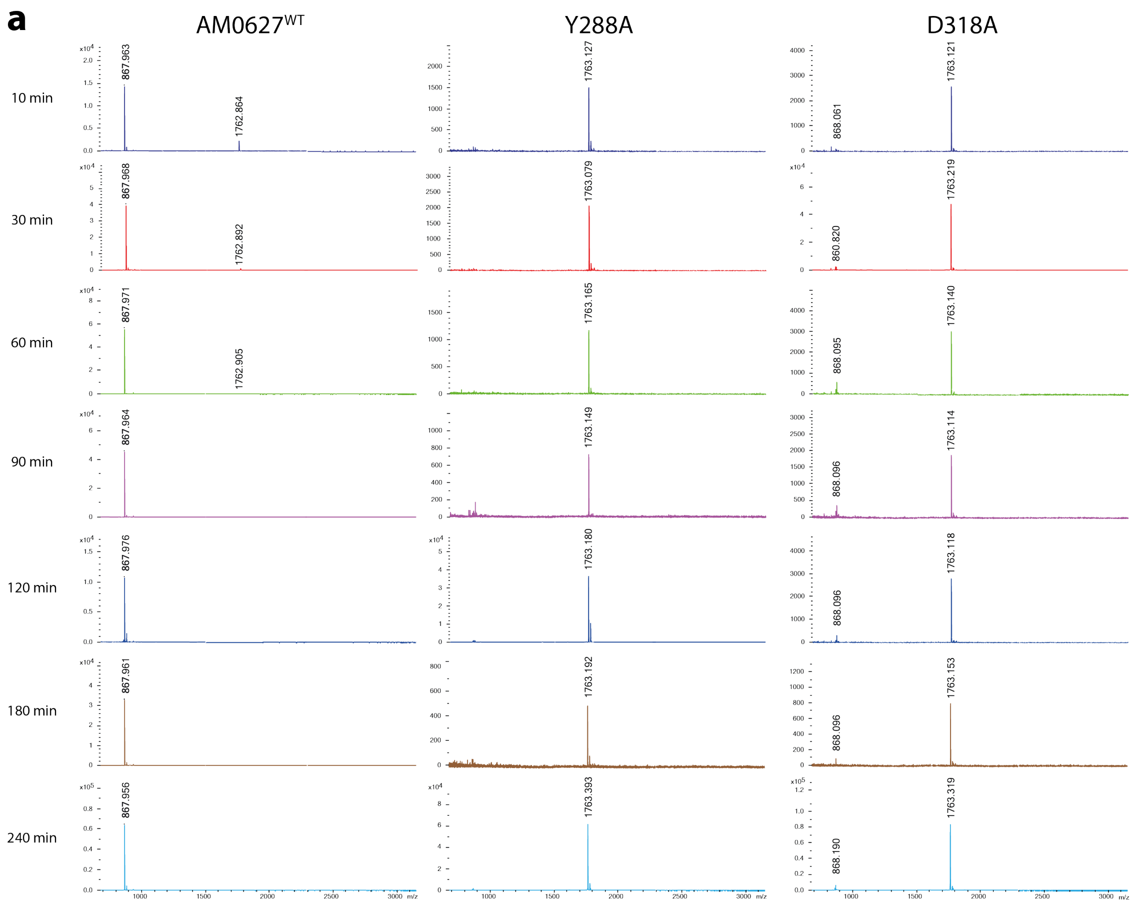

Supplementary Fig. 3. MALDI-TOF analysis of in vitro cleavage of wt AM0627$^{A21-E506}$ and mutants against P1. 600 ng (0.4 μM) of the wt AM0627$^{A21-E506}$ and mutants was used in all the experiments. A peak intesity of substrate (S) and product (P) were semi-quantified and the remaining substrate ratio were estimated. See below for the MALDI-TOF experiments for the rest of the mutants.

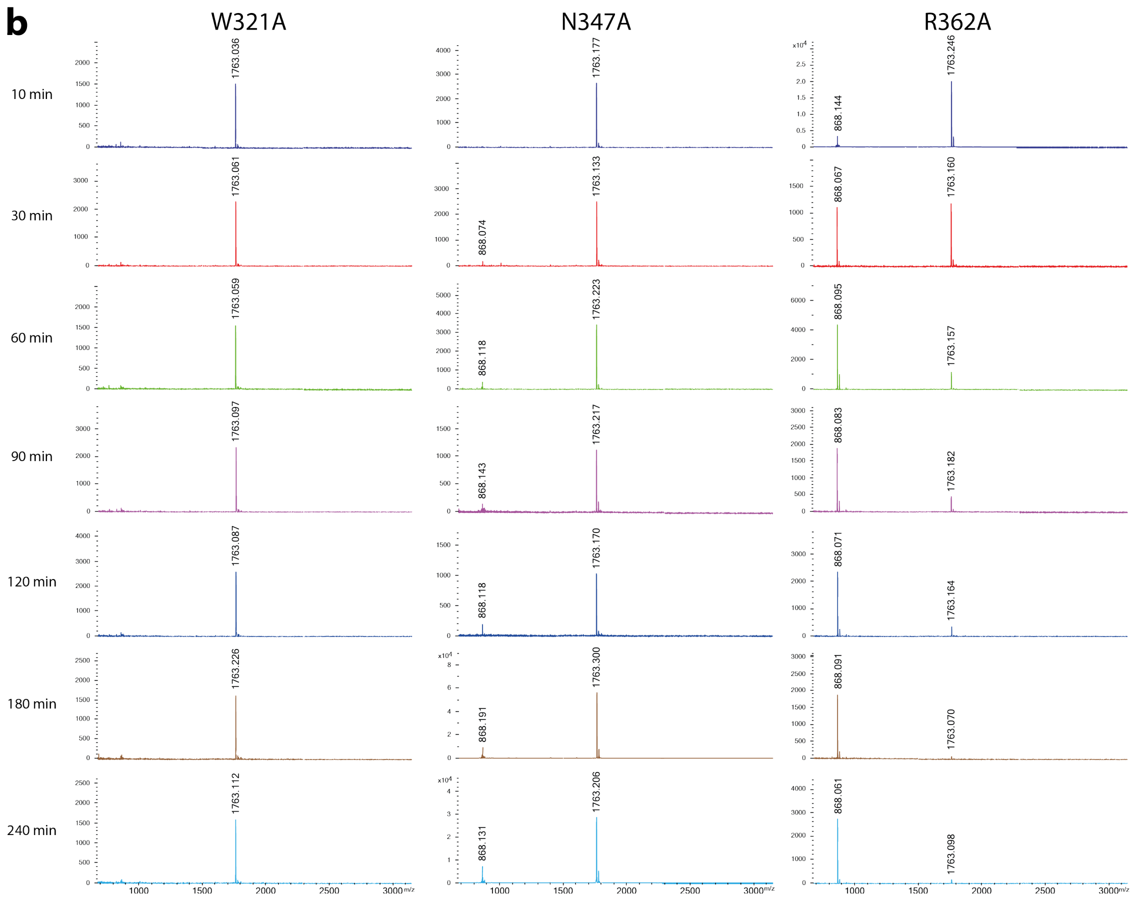

Supplementary Fig. 3.

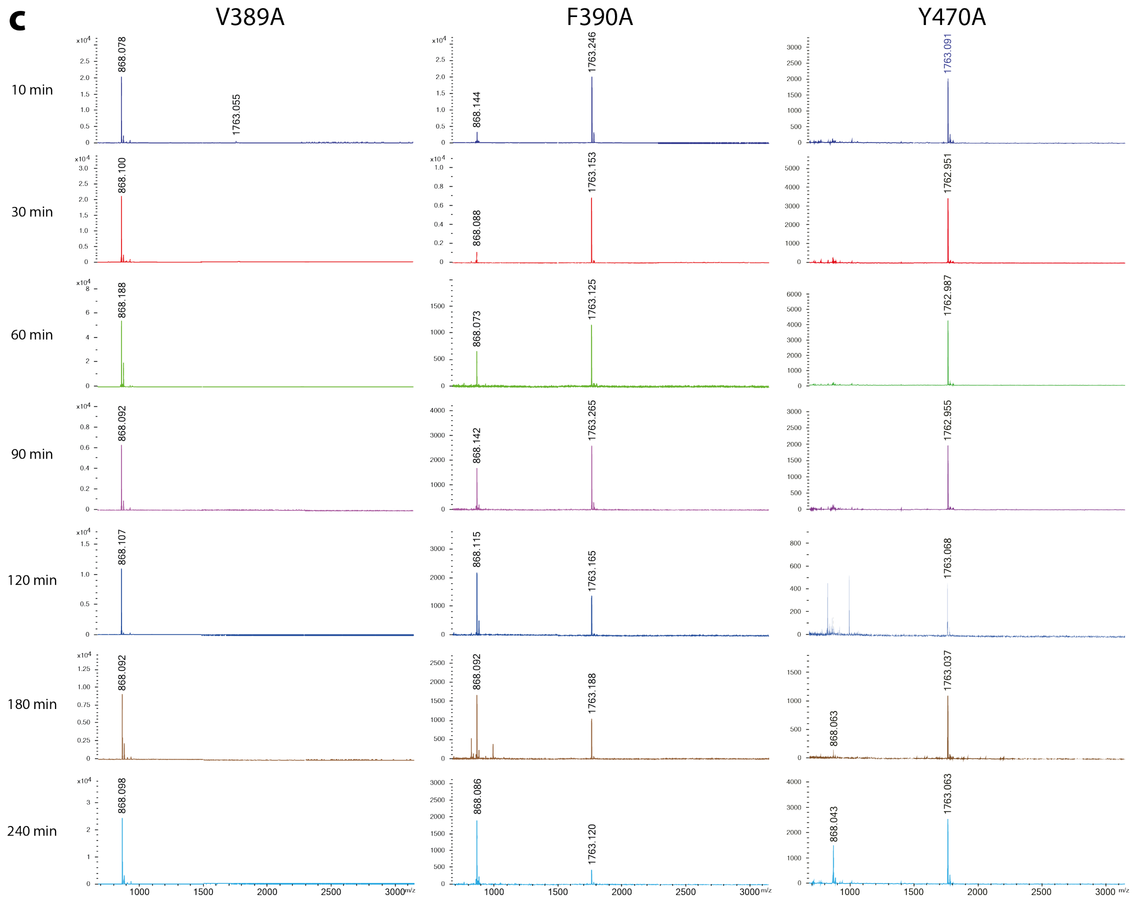

Supplementary Fig. 3.

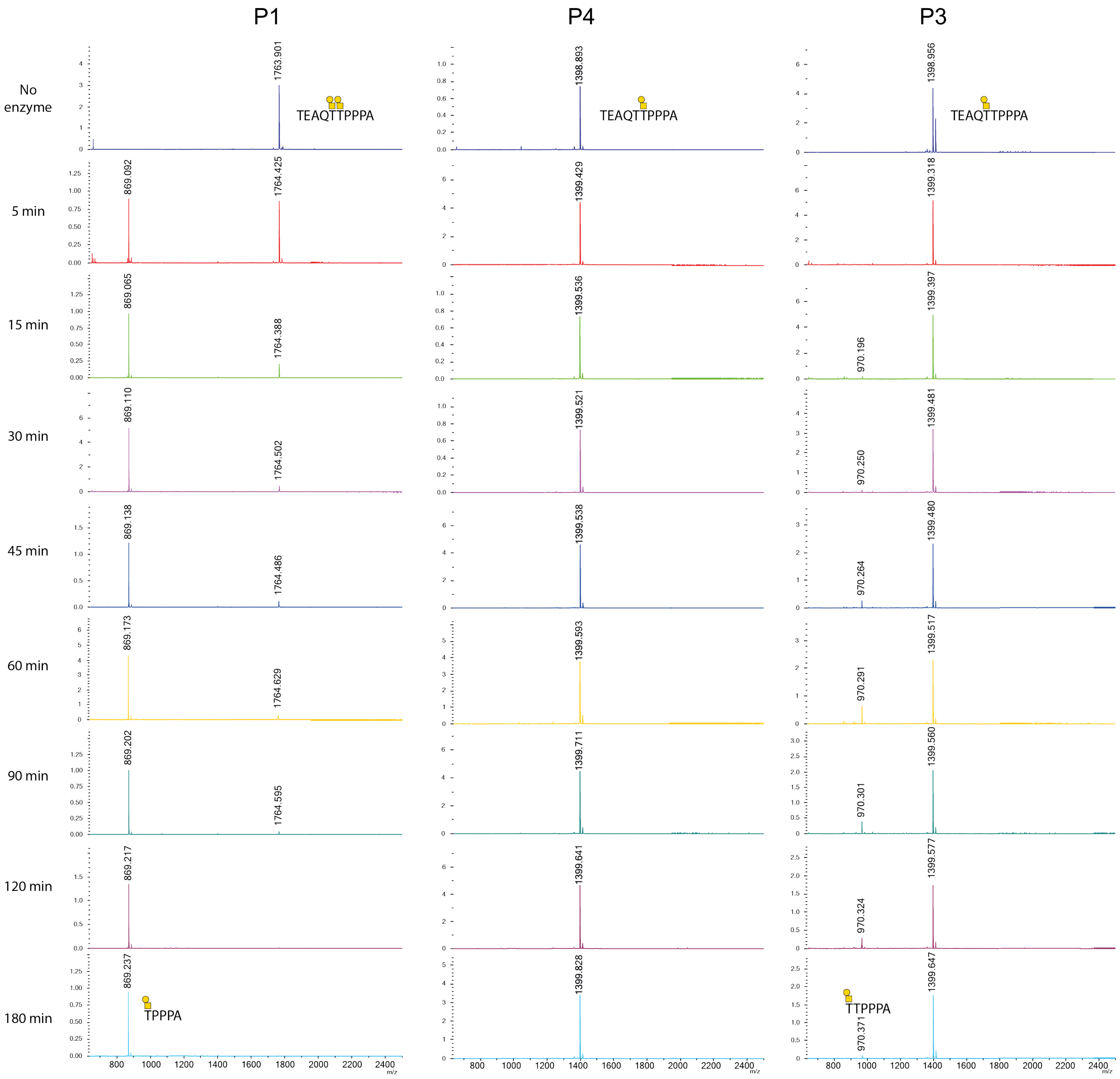

Supplementary Fig. 4. MALDI-TOF analysis of in vitro cleavage of wt AM0627$^{A21\text{-}E506}$ against a series of (glyco)peptides. 600 ng (0.4 μM) of the wt AM0627$^{A21\text{-}E506}$ was used in all the experiments. A peak intesity of substrate (S) and product (P) were semi-quantified and the remaining substrate ratio were estimated.

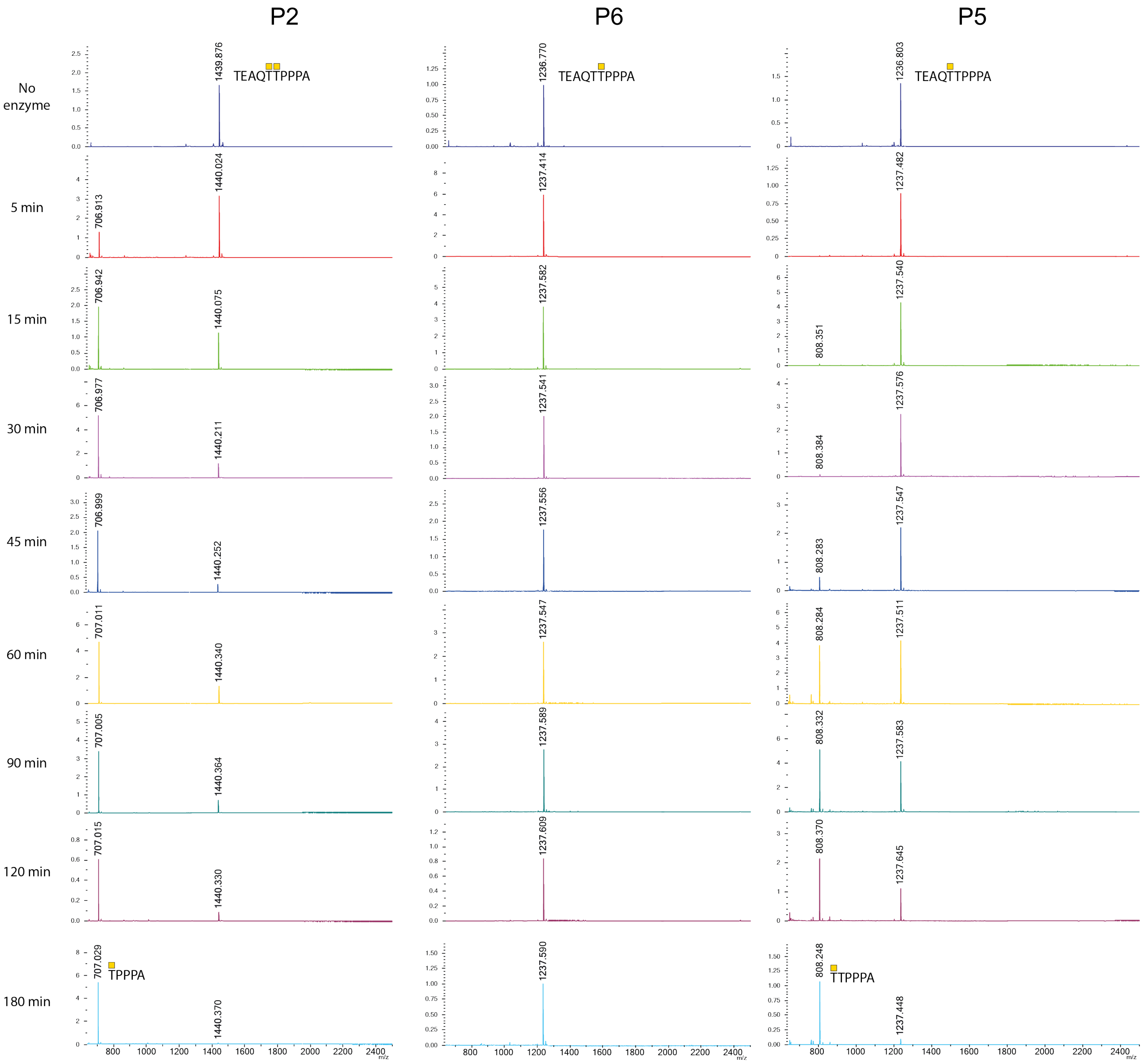

Supplementary Fig. 4.

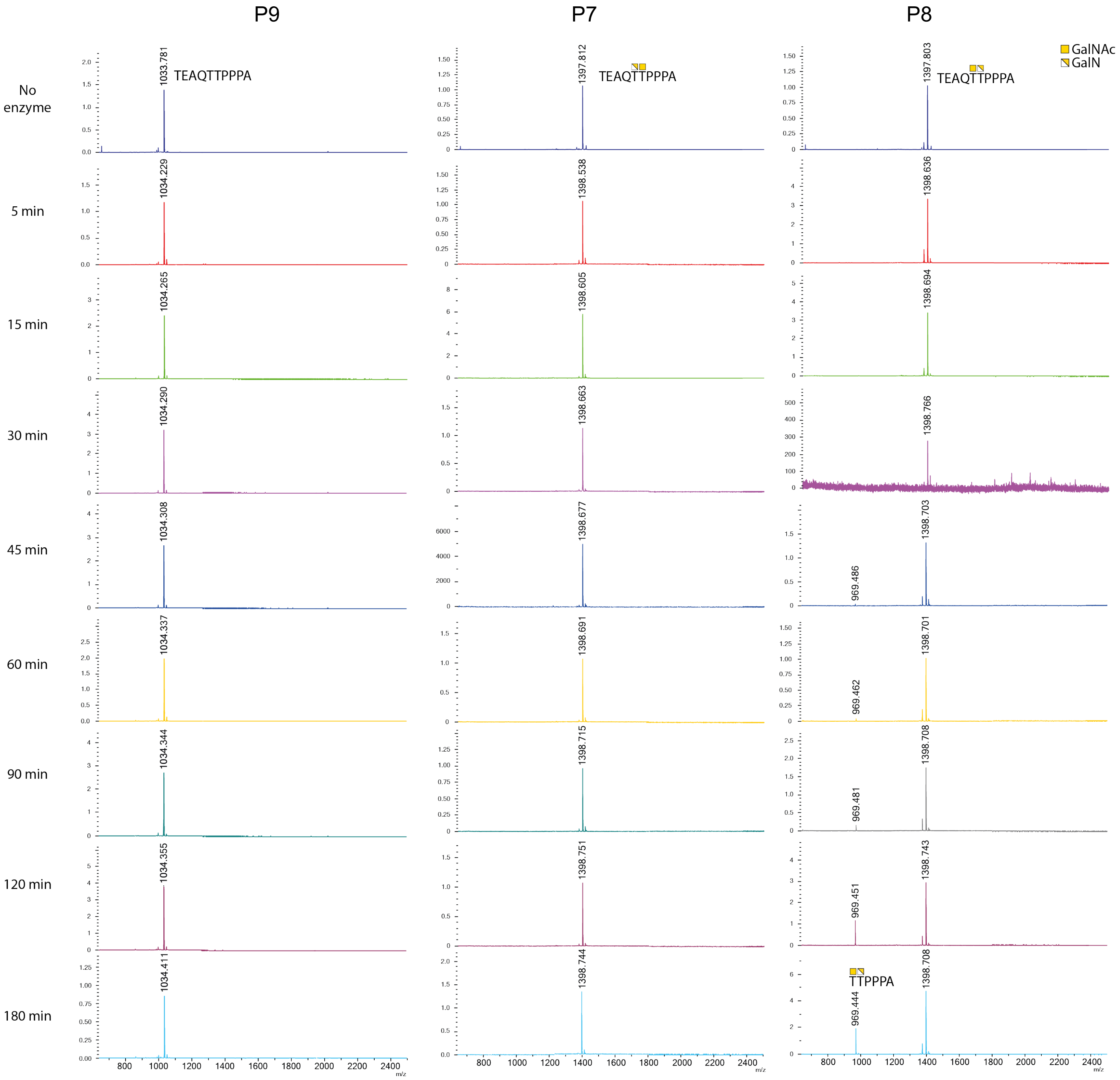

Supplementary Fig. 4.

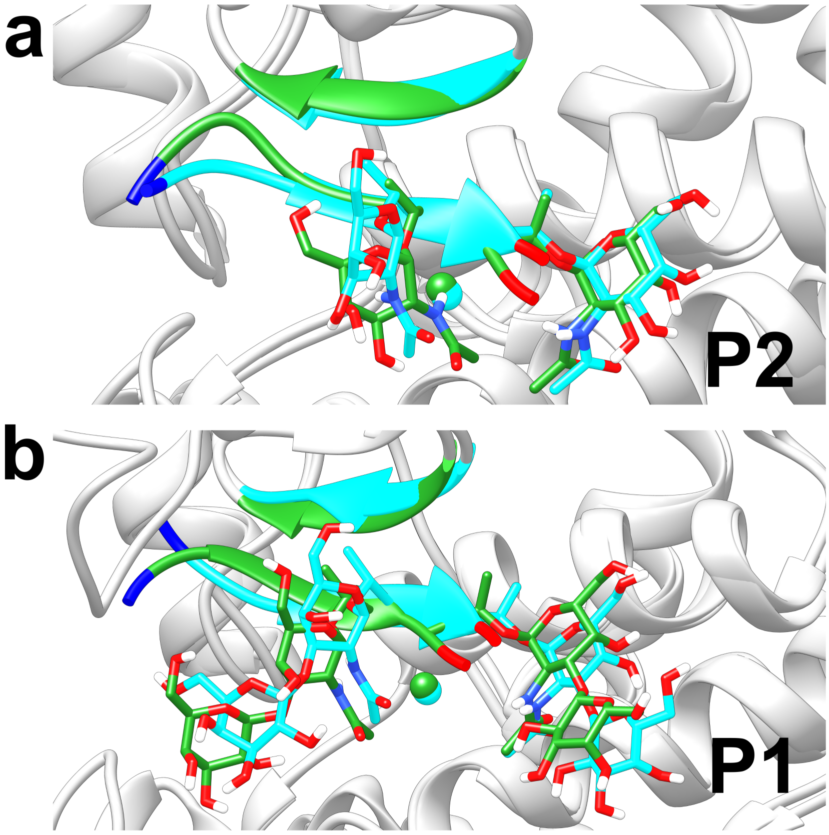

Supplementary Figure 5. a and **b**, A closer view of the complexes of AM0627 and glycopeptides **P2** and **P1**, respectively, as showed in **Figs. 4e**,**f**. The initial positions of the glycopeptide, the β-sheet (Met289-Asp292) and Zn^{2+} are shown in green, while the final positions (after one of the 500 ns replicas) are shown in cyan. The N- and C-termini of the glycopeptides are colored in blue and red, respectively. The Gal and GalNAC molecules are showed in stick.

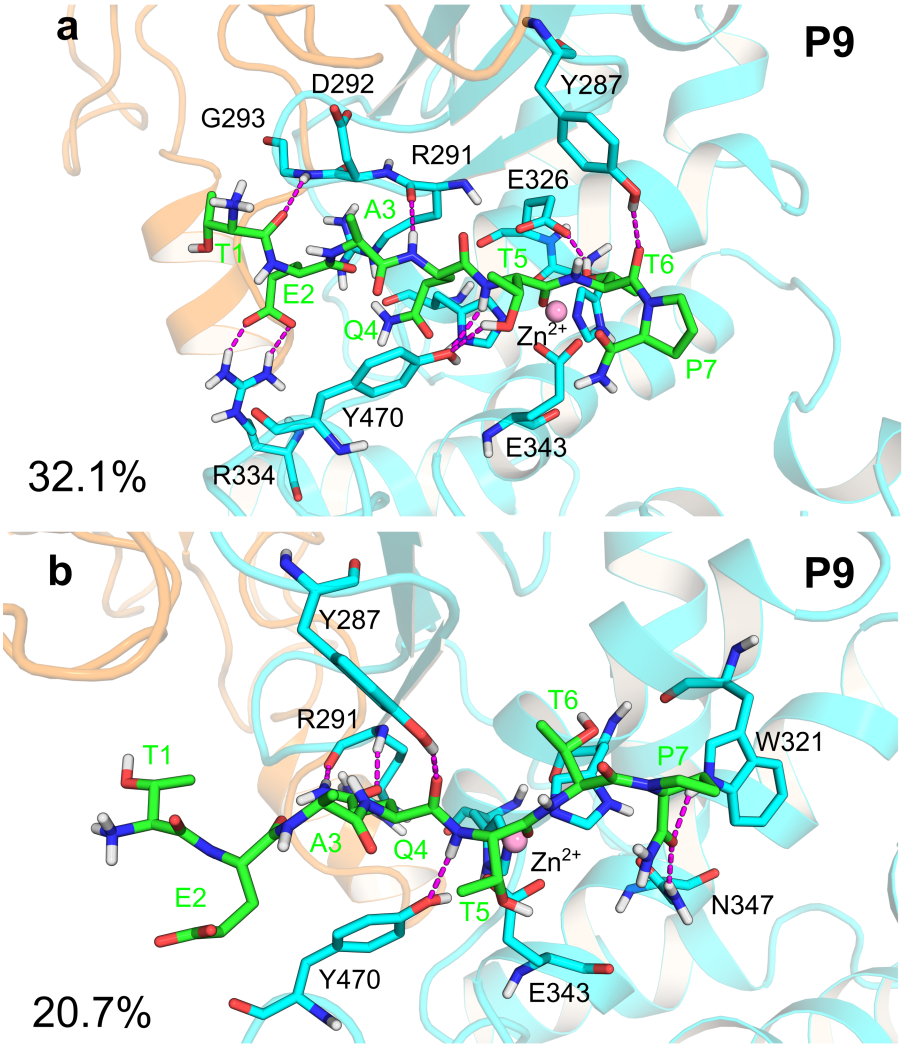

Supplementary Figure 6. a and **b**, **Representative configurations of the two most populated clusters of the AM0627-P9 complex**. The clustering was done over the three replicas based on the RMSD of the peptide backbone atoms with a cutoff of 0.5 Å after an alignment over the protease backbone atoms using CPPTRAJ of Amber 20. The population of the clusters are also labelled.

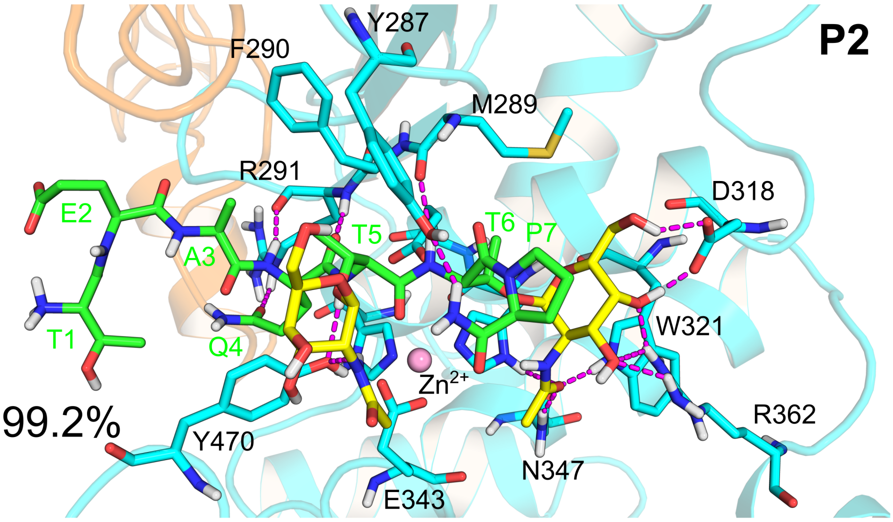

Supplementary Figure 7. Representative configurations of the most populated cluster of the AM0627-P2 complex. The clustering was done over the three replicas based on the RMSD of the peptide backbone atoms with a cutoff of 0.5 Å after an alignment over the protease backbone atoms using CPPTRAJ of Amber 20. The population of the cluster is labelled.

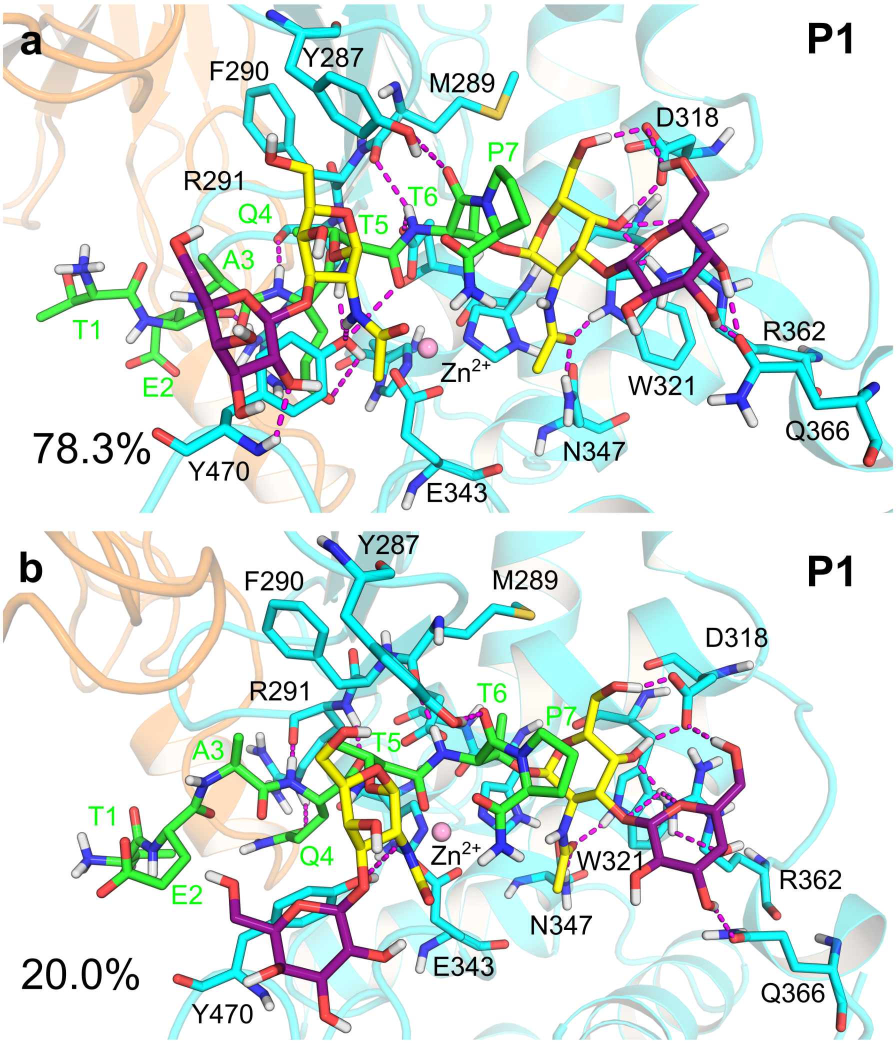

Supplementary Figure 8. a and b, Representative configurations of the two most populated clusters of the AM0627-P1 complex. The clustering was done over the three replicas based on the RMSD of the peptide backbone atoms with a cutoff of 0.5 Å after an alignment over the protease backbone atoms using CPPTRAJ of Amber 20. The population of the clusters are also labelled.

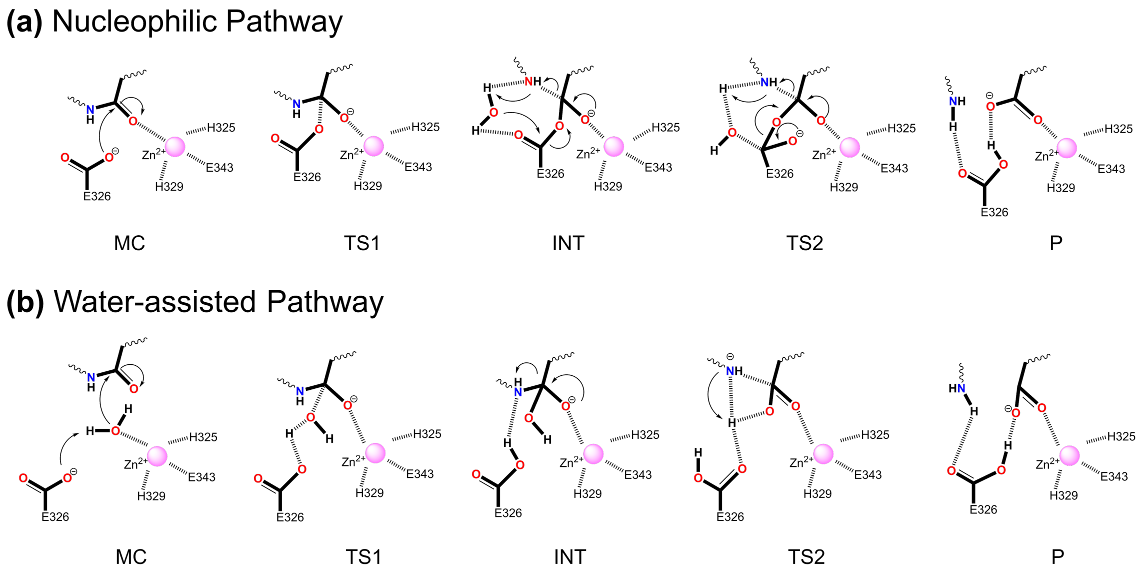

Supplementary Figure 9. Scheme of the two possible reaction pathways or mechanisms proposed for Zn-metalloproteases. a, The nucleophilic pathway, and **b**, the water-assisted pathway, for peptide bond hydrolysis catalyzed by Zn-metalloproteases. Note that the AM0627 active site is depicted for illustration purposes.

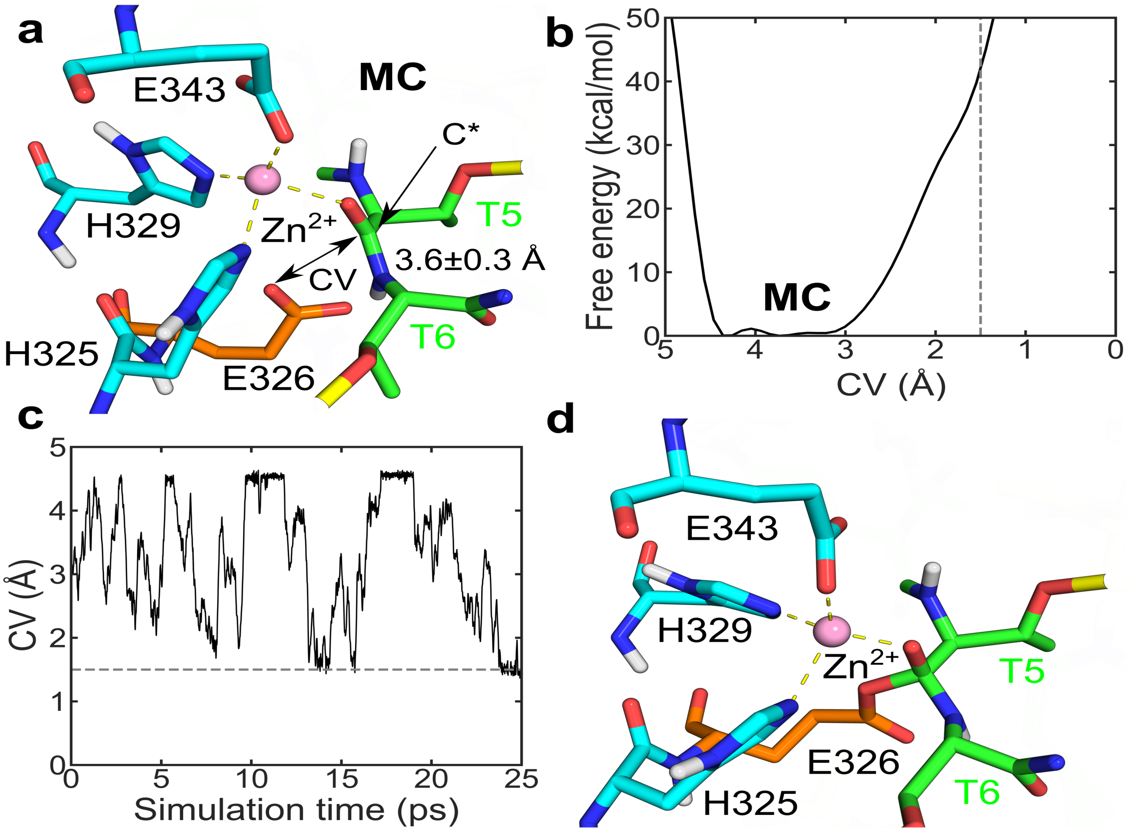

Supplementary Figure 10. The QM/MM metadynamics simulation of the first reaction step considering the direct nucleophilic pathway. a, A representative snapshot of the Michaelis complex, the average distance between the catalytic carboxylate oxygen and the carbonyl carbon during the QM/MM MD equilibration is indicated. **b**, Free energy profile of the reaction leading to an unstable state in which E326 is coordinated to T5 (dashed line). **c**, Evolution of the collective variable (CV = O_{E326}-C^{*}_{T5} distance) during the metadynamics simulation. **d**, A representative snapshot of the unstable state resulting from the nucleophilic attack.

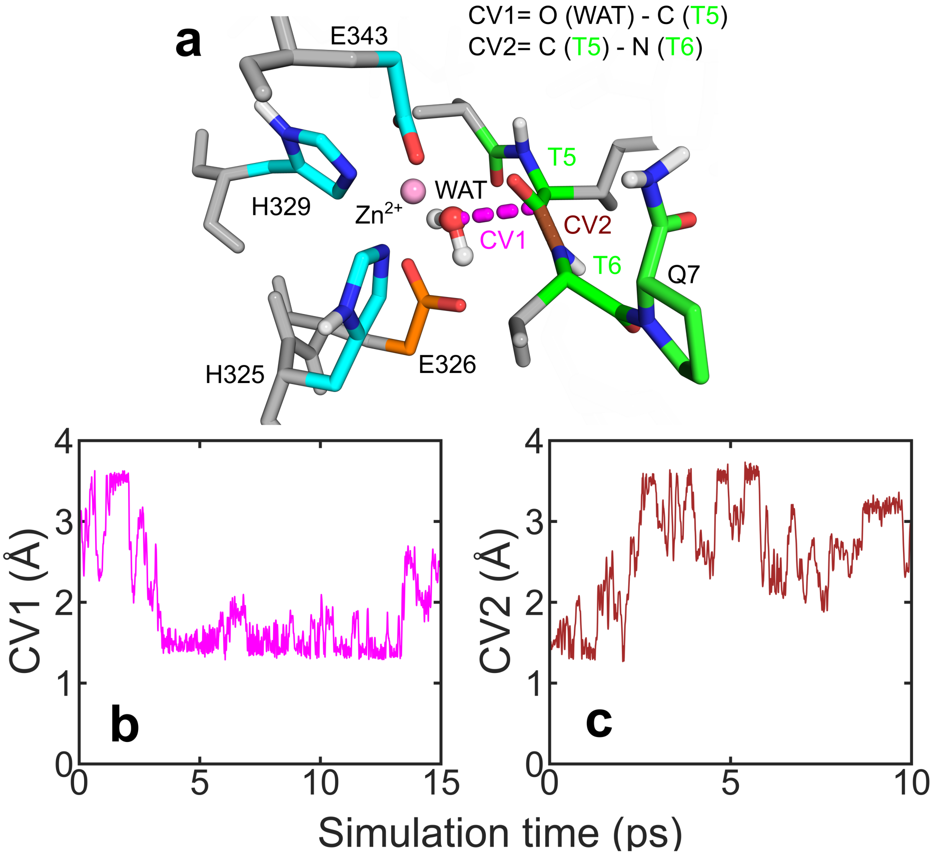

Supplementary Figure 11. The QM/MM simulation of the water-assisted mechanisms. **a**, The QM region and collective variables (CVs) used in the QM/MM simulations. The atoms in grey are in the MM region while atoms in non-grey are in the QM region. Non-polar hydrogen atoms are hidden. **b**, Evolution of the collective variables CV1, and **c**, CV2 during the QM/MM metadynamics simulations.

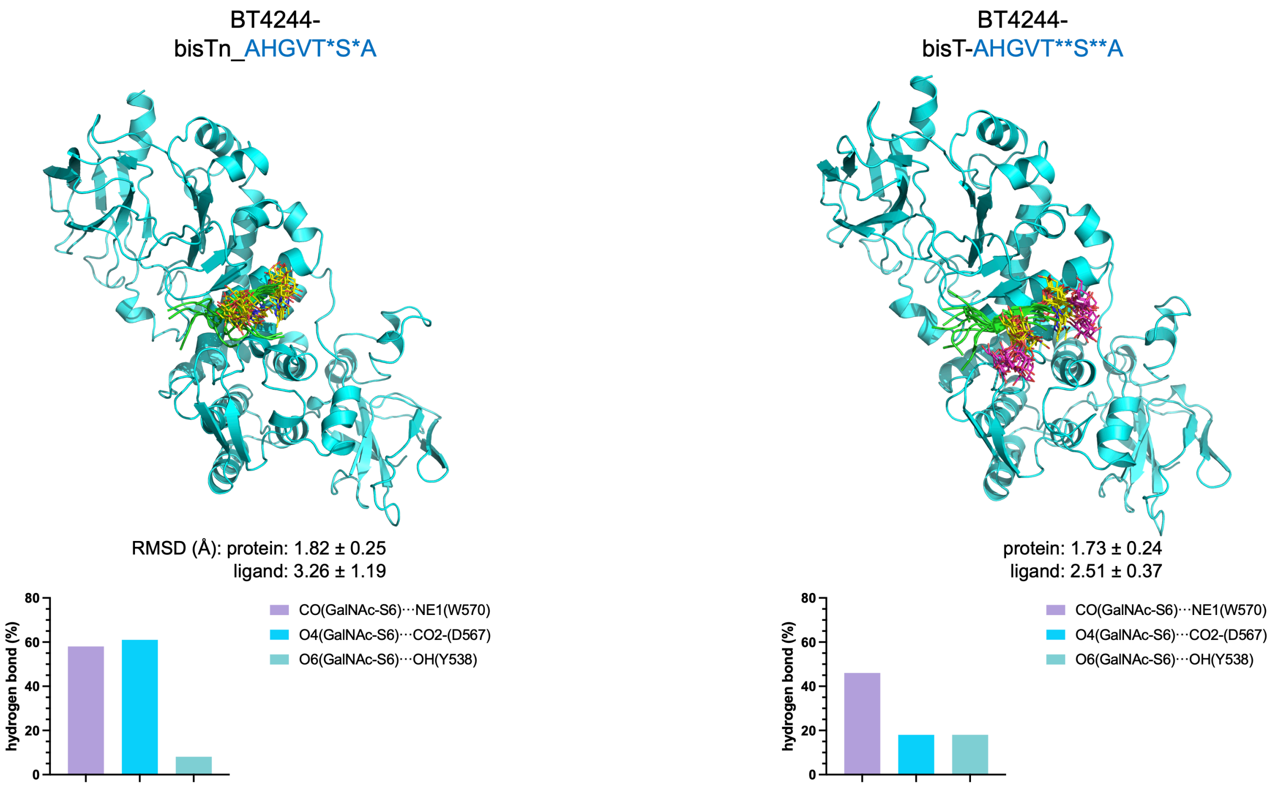

Supplementary Figure 12. Structural ensembles derived from 0.5 µs MD simulations, together with the RMSD values for the protein (backbone atoms) and the ligand (heavy atoms). The population (%) of the hydrogen bonds found in the simulations between the sugar moieties of the ligands (bis-Tn and bis-T) and BT4244 are also shown. The MD simulations clearly show that the complexes are stable over the entire trajectory.

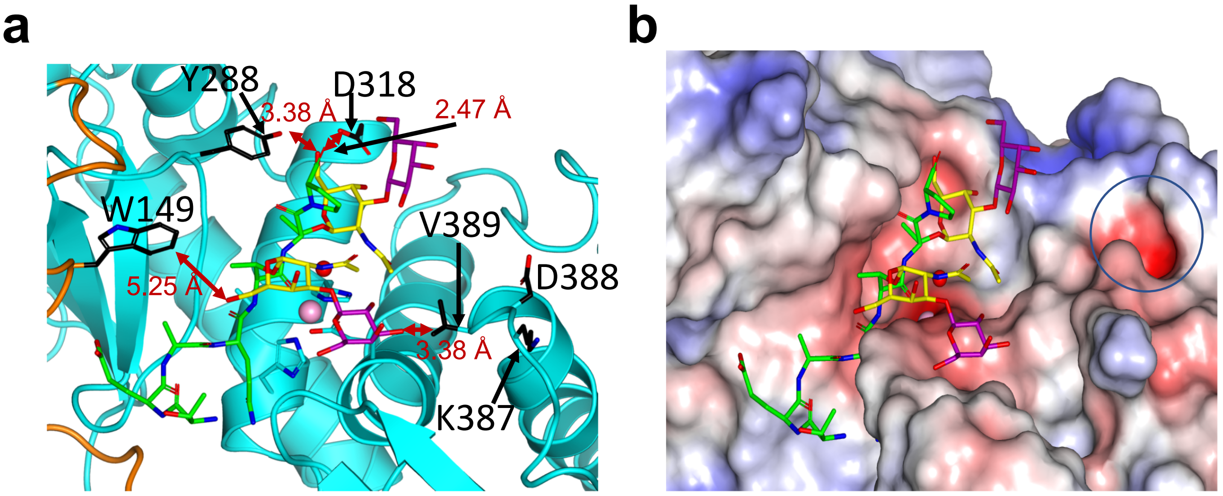

Supplementary Figure 13. a, Active site of AM0627^{E326A} complexed to P1 and showing the distances between the GalNAc OH6 and Gal OH3 at G1/G1` and G2, respectively, with the nearby residues from the mucinase. **b,** Electrostatic surface representation of AM0627^{E326A} (scale bar ranged from -5 kTe-1 to +5 kTe-1). The circle indicates a negatively charge surface that might disrupt the binding of Sia, explaining why AM0627 might not cleave on glycoproteins containing mSTa. Overall, our structural analysis might explain why AM0627 will not cleave on mSTa, mSTb, STn antigen and dST.

Supplementary Table 1. Data collection and refinement statistics.

	$AM0627^{E326A}$ (71-506) in complex with P1 and Zn^{2+}	$AM0627^{E326A}$ (71-506) in complex with P1 and Zn^{2+}
**Data collection**		
Space group	$P2_12_12_1$	$P2_12_12_1$
Wavelength (Å)	1.2817	0.9792
Cell dimensions		
a, *b*, *c* (Å)	74.05, 82.22, 173.81	73.14, 82.69, 172.61
α, β, γ (°)	90, 90, 90	90, 90, 90
Number of protein molecules per asymmetric unit	2	2
Resolution (Å)	173.81-1.90 (2.00-1.90)*	172.61-1.50 (1.58-1.50)*
R_{merge}	0.153 (2.140)	0.058 (1.092)
$I / \sigma I$	13.9 (2.5)	12.6 (1.6)
Completeness (%)	100 (100)	100 (100)
Redundancy	24.8 (22)	7.3 (7.2)
Mn(I) half-set correlation CC(1/2)	0.999 (0.781)	0.999 (0.624)
**Refinement**		
Resolution (Å)	1.90	1.50
Total number of observations reflections	2091294	1229955
Total number unique reflections	84443	167774
R_{work} / R_{free}		0.167/0.1821
No. atoms		
Protein		7048
TAg glycopeptide		211
Zn^{+2}		2
Waters		769
Glycerol		72
B-factors ($Å^2$)		
Protein		28.74
P1		52.30
Zn^{2+}		20.85
Waters		39.87
Glycerol		55.63
R.m.s. deviations		
Bond lengths (Å)		0.0083
Bond angles (°)		1.5532

One crystal was used to determine the crystal structure. *Values in parentheses are for highest-resolution shell.
