# Supplementary material for: Structural and mechanistic insights into the cleavage of clustered O-glycan patches-containing glycoproteins by mucinases of the human gut
Source: Nat Commun. 2022 Jul 26;13:4324. doi: 10.1038/s41467-022-32021-9 (PMC9325726; doi:10.1038/s41467-022-32021-9)
Supplement: Supplementary file 2 — Reporting Summary [file 41467_2022_32021_MOESM2_ESM.pdf]

nature portfolio

Corresponding author(s): Ramon Hurtado-Guerrero
Carme Rovira

Last updated by author(s): Jul 1, 2022

Reporting Summary

Nature Portfolio wishes to improve the reproducibility of the work that we publish. This form provides structure for consistency and transparency in reporting. For further information on Nature Portfolio policies, see our Editorial Policies and the Editorial Policy Checklist.

Statistics

For all statistical analyses, confirm that the following items are present in the figure legend, table legend, main text, or Methods section.

n/a	Confirmed	
☐	☒	The exact sample size (n) for each experimental group/condition, given as a discrete number and unit of measurement
☐	☒	A statement on whether measurements were taken from distinct samples or whether the same sample was measured repeatedly
☒	☐	The statistical test(s) used AND whether they are one- or two-sided<br>*Only common tests should be described solely by name; describe more complex techniques in the Methods section.*
☒	☐	A description of all covariates tested
☒	☐	A description of any assumptions or corrections, such as tests of normality and adjustment for multiple comparisons
☒	☐	A full description of the statistical parameters including central tendency (e.g. means) or other basic estimates (e.g. regression coefficient) AND variation (e.g. standard deviation) or associated estimates of uncertainty (e.g. confidence intervals)
☒	☐	For null hypothesis testing, the test statistic (e.g. F, t, r) with confidence intervals, effect sizes, degrees of freedom and P value noted<br>*Give P values as exact values whenever suitable.*
☒	☐	For Bayesian analysis, information on the choice of priors and Markov chain Monte Carlo settings
☒	☐	For hierarchical and complex designs, identification of the appropriate level for tests and full reporting of outcomes
☒	☐	Estimates of effect sizes (e.g. Cohen's d, Pearson's r), indicating how they were calculated

Our web collection on statistics for biologists contains articles on many of the points above.

Software and code

Policy information about availability of computer code

Data collection	The protein crystals were diffracted and collected on beamline XALOC at ALBA synchrotron (Barcelona).
Data analysis	Crystallography was processed as described in Methods.<br>For X-ray data, we used CCP4 software 7.0.078, XDS version 5 2021, Refmac5, SHELXD, Procheck, PYMOL 2.4.2 and COOT 0.9.6.<br>For Molecular Dynamics simulations, we used AMBER 20 package.<br>For QM/MM metadynamics, we used CP2K v7.1 interfaced with PLUMED v2.5.<br>The kinetics of our enzyme was followed by MALDI-TOF MS.<br>All the software used in this manuscript are published and references to the different programs are cited in the manuscript.

For manuscripts utilizing custom algorithms or software that are central to the research but not yet described in published literature, software must be made available to editors and reviewers. We strongly encourage code deposition in a community repository (e.g. GitHub). See the Nature Portfolio guidelines for submitting code & software for further information.

Data

Policy information about availability of data

All manuscripts must include a data availability statement. This statement should provide the following information, where applicable:

- Accession codes, unique identifiers, or web links for publicly available datasets
- A description of any restrictions on data availability
- For clinical datasets or third party data, please ensure that the statement adheres to our policy

The crystal structure of the AM0627^{E326A}-**P1**-Zn^{2+} complex was deposited at the RCSB PDB with accession code 7YX8 [https://doi.org/10.2210/pdb7YX8/pdb]. Previously published PDB structures used in this study are available under the accession codes: 5KD8 [https://doi.org/10.2210/pdb5KD8/pdb], 5KDU [https://doi.org/10.2210/pdb5KDU/pdb], 6XT1 [https://doi.org/10.2210/pdb6XT1/pdb], 5KDX [https://doi.org/10.2210/pdb5KDX/pdb], 6Z2P [https://doi.org/10.2210/pdb6Z2P/pdb] and 7SCI [https://doi.org/10.2210/pdb7SCI/pdb].The trajectory files of the classical MD simulation and QM/MM metadynamics simulations have been deposited to Zenodo at https://doi.org/10.5281/zenodo.6521230. Other data are available from the corresponding author upon request. Source data are provided with this paper.

Field-specific reporting

Please select the one below that is the best fit for your research. If you are not sure, read the appropriate sections before making your selection.

- [x] Life sciences
- [ ] Behavioural & social sciences
- [ ] Ecological, evolutionary & environmental sciences

For a reference copy of the document with all sections, see nature.com/documents/nr-reporting-summary-flat.pdf

Life sciences study design

All studies must disclose on these points even when the disclosure is negative.

Sample size	No statistical methods were used to predetermine sample size. We used two independent experiments for mass-spec experiments.<br>For molecular dynamics simulations, we performed the experiments in triplicate.<br>All attempts at replication were successful.
Data exclusions	No data was excluded from the experiments.
Replication	We used two independent experiments for mass-spec experiments.<br>For molecular dynamics simulations, we performed the experiments in triplicate.<br>All attempts at replication were successful.
Randomization	This is not relevant for this study.
Blinding	Binding s is not relevant for this study.

Reporting for specific materials, systems and methods

We require information from authors about some types of materials, experimental systems and methods used in many studies. Here, indicate whether each material, system or method listed is relevant to your study. If you are not sure if a list item applies to your research, read the appropriate section before selecting a response.

Materials & experimental systems

n/a	Involved in the study	
☒	☐	Antibodies
☐	☒	Eukaryotic cell lines
☒	☐	Palaeontology and archaeology
☒	☐	Animals and other organisms
☒	☐	Human research participants
☒	☐	Clinical data
☒	☐	Dual use research of concern

Methods

n/a	Involved in the study	
☒	☐	ChIP-seq
☒	☐	Flow cytometry
☒	☐	

Eukaryotic cell lines

Policy information about cell lines

Cell line source(s)	Glycoengineered HEK293 cell lines with O-glycan designs for Tn (knockout (KO) C1GALT1), core1 (KO GCNT1, ST3GAL1/2, ST6GALNAC2/3/4), mono-sialylT (mSTa) (KO GCNT1, ST6GALNACT2/3/4) and wildtype HEK293WT were used for the stable expression of MUC1 TR reporter and are available as part of the cell-based glycan array resource. The original HEK293 was purchased from GIBCO.
Authentication	No specific authentication of cell lines used apart from separate handling of original obtained vials throughout entire project. Each individual engineered HEK293 clones were confirmed multiple times by HEK293 gene specific IDAA and Sanger sequencing in the target gene area(s).
Mycoplasma contamination	A representative set of growing cell lines in the lab selected randomly is subjected to mycoplasma screening bi-monthly, and within the last 10 yrs no infected cells have been found.
Commonly misidentified lines (See ICLAC register)	None of the cell lines used are listed in the ICLAC database.
